# Herpes Simplex Virus 1-Induced Ferroptosis Contributes to Viral Encephalitis

**DOI:** 10.1128/mbio.02370-22

**Published:** 2022-12-12

**Authors:** Xi-Qiu Xu, Tongran Xu, Wenting Ji, Chong Wang, Yujie Ren, Xiaobei Xiong, Xi Zhou, Shu-Hai Lin, Yi Xu, Yang Qiu

**Affiliations:** a Guangzhou Institute of Pediatrics, Guangzhou Women and Children’s Medical Center, Guangzhou, Guangdong, China; b State Key Laboratory of Virology, Wuhan Institute of Virology, Chinese Academy of Sciences, Wuhan, Hubei, China; c State Key Laboratory of Cellular Stress Biology, School of Life Sciences, Faculty of Medicine and Life Sciences, Xiamen University, Xiamen, China; d School of Life Sciences, Division of Life Sciences and Medicine, University of Science and Technology of China, Hefei, Anhui, China; The University of North Carolina at Chapel Hill

**Keywords:** ferroptosis, HSV-1, Nrf2-Keap1, PTGS2/PGE_2_, viral encephalitis

## Abstract

Herpes simplex virus 1 (HSV-1) is a DNA virus belonging to the family *Herpesviridae*. HSV-1 infection causes severe neurological disease in the central nervous system (CNS), including encephalitis. Ferroptosis is a nonapoptotic form of programmed cell death that contributes to different neurological inflammatory diseases. However, whether HSV-1 induces ferroptosis in the CNS and the role of ferroptosis in viral pathogenesis remain unclear. Here, we demonstrate that HSV-1 induces ferroptosis, as hallmarks of ferroptosis, including Fe^2+^ overload, reactive oxygen species (ROS) accumulation, glutathione (GSH) depletion, lipid peroxidation, and mitochondrion shrinkage, are observed in HSV-1-infected cultured human astrocytes, microglia cells, and murine brains. Moreover, HSV-1 infection enhances the E3 ubiquitin ligase Keap1 (Kelch-like ECH-related protein 1)-mediated ubiquitination and degradation of nuclear factor E2-related factor 2 (Nrf2), a transcription factor that regulates the expression of antioxidative genes, thereby disturbing cellular redox homeostasis and promoting ferroptosis. Furthermore, HSV-1-induced ferroptosis is tightly associated with the process of viral encephalitis in a mouse model, and the ferroptosis-activated upregulation of prostaglandin-endoperoxide synthase 2 (PTGS2) and prostaglandin E_2_ (PGE_2_) plays an important role in HSV-1-caused inflammation and encephalitis. Importantly, the inhibition of ferroptosis by a ferroptosis inhibitor or a proteasome inhibitor to suppress Nrf2 degradation effectively alleviated HSV-1 encephalitis. Together, our findings demonstrate the interaction between HSV-1 infection and ferroptosis and provide novel insights into the pathogenesis of HSV-1 encephalitis.

## INTRODUCTION

Herpes simplex virus 1 (HSV-1), an enveloped double-stranded DNA virus belonging to the genus *Simplexvirus* of the family *Herpesviridae*, is a ubiquitous pathogen that commonly infects humans (1). It has been estimated that 3.7 billion people under 50 years of age have HSV-1 infection globally, and HSV-1 is the most frequently identified cause of sporadic encephalitis worldwide (2). HSV-1 encephalitis (HSE) causes severe neuroinflammation and impairment of neurological functions in the central nervous system (CNS), resulting in a wide spectrum of clinical manifestations such as cognitive dysfunction, personality changes, aphasia, and seizures, and the mortality and morbidity rates of HSE are high, with the majority of surviving patients developing severe neurological sequelae despite the use of antiviral therapy (3). Thus far, the mechanisms underlying the pathogenesis of HSV-1-induced neurological diseases have not been completely elucidated.

Ferroptosis is a novel nonapoptotic form of programmed cell death that depends on the formation and accumulation of iron-mediated lipid free radical (4). Ferroptosis is characterized by the generation of excessive intracellular lipid reactive oxygen species (ROS) and causes fatal cell lipid peroxidation when the cellular antioxidant capacity is reduced (5). Based on this, ferroptosis sensitivity is tightly regulated by the biological processes involved in maintaining redox homeostasis, including the production of ROS and the biosynthesis of glutathione (GSH), the main intracellular small-molecule antioxidant. Glutathione peroxidase 4 (GPX4) utilizes reduced GSH to convert lipid hydroperoxides to lipid alcohols via oxidizing GSH to oxidized glutathione (GSSG), thereby preventing the lipid peroxidation of the cell membrane and inhibiting ferroptosis (6). In contrast, the depletion of intracellular GSH results in the massive accumulation of fatal ROS (7).

The transcription factor nuclear factor E2-related factor 2 (Nrf2) is a major regulator responsible for the expression of a series of antioxidant genes involved in antioxidant response pathways, including iron-dependent ROS production, GSH homeostasis, and GPX4 activity (8). Therefore, Nrf2 is considered to be an important negative regulator for controlling the process of ferroptosis. The activity of Nrf2 is regulated by Kelch-like ECH-related protein 1 (Keap1) and the ubiquitin (Ub)-proteasome system (UPS) (9). Under homeostatic conditions, Keap1 interacts with Nrf2 and directs Nrf2 degradation via the UPS. In response to oxidative stress, Nrf2 dissociates from Keap1 and translocates to the nucleus, leading to the increased expression of antioxidant-related genes (10). Moreover, noncanonical p62-mediated autophagic degradation has also been found to regulate Nrf2 (11).

Accumulating evidence shows that ferroptosis plays an important role in neurological diseases since the brain has the highest level of polyunsaturated fatty acids, which are the precursors of lipid peroxide (12). For instance, ferroptosis has been observed in dopaminergic neurons in Parkinson’s disease and other neurodegenerative diseases (13, 14). Lipid peroxidation and GSH depletion are tightly associated with neurological diseases such as neurotrauma, stroke, and neurodegeneration (15). Moreover, the inhibition of neuronal ferroptosis has been shown to protect brains from spontaneous intracerebral hemorrhage (16).

Moreover, common features of ferroptosis, including iron overload, ROS accumulation, and GSH depletion, have been observed during infections by various viruses, suggesting the possible contribution of ferroptosis to viral replication and pathogenesis. Previous studies have shown that HSV-1 infection induces the production of ROS in murine neural cells (17, 18). Furthermore, canonical antioxidants that are considered the inhibitors of ferroptosis have been found to suppress HSV-1 replication (19). However, whether HSV-1 induces ferroptosis in neural cells remains elusive. More importantly, it is unclear whether and how ferroptosis functions in HSV-1-caused neurological diseases, which therefore hinders the understanding of the physiological impact of ferroptosis on HSV-1 neuropathogenesis and the therapeutic potential of inhibiting ferroptosis in viral diseases.

In this study, we determined that HSV-1 induced ferroptosis in human astrocytoma and microglial cells and *in vivo*, and we applied a mouse model to assess its physiological impact on the pathogenesis of HSV-1 infection. We report that HSV-1-induced ferroptosis and HSV-1-enhanced Nrf2 degradation contribute to the development of ferroptosis. HSV-1-caused encephalitis is tightly correlated with virus-induced ferroptosis, and the inhibition of ferroptosis effectively alleviates HSV-1-caused neuropathogenic damage.

## RESULTS

### HSV-1 induces ferroptosis in human astrocytes and microglia cells.

**FIG 1:**
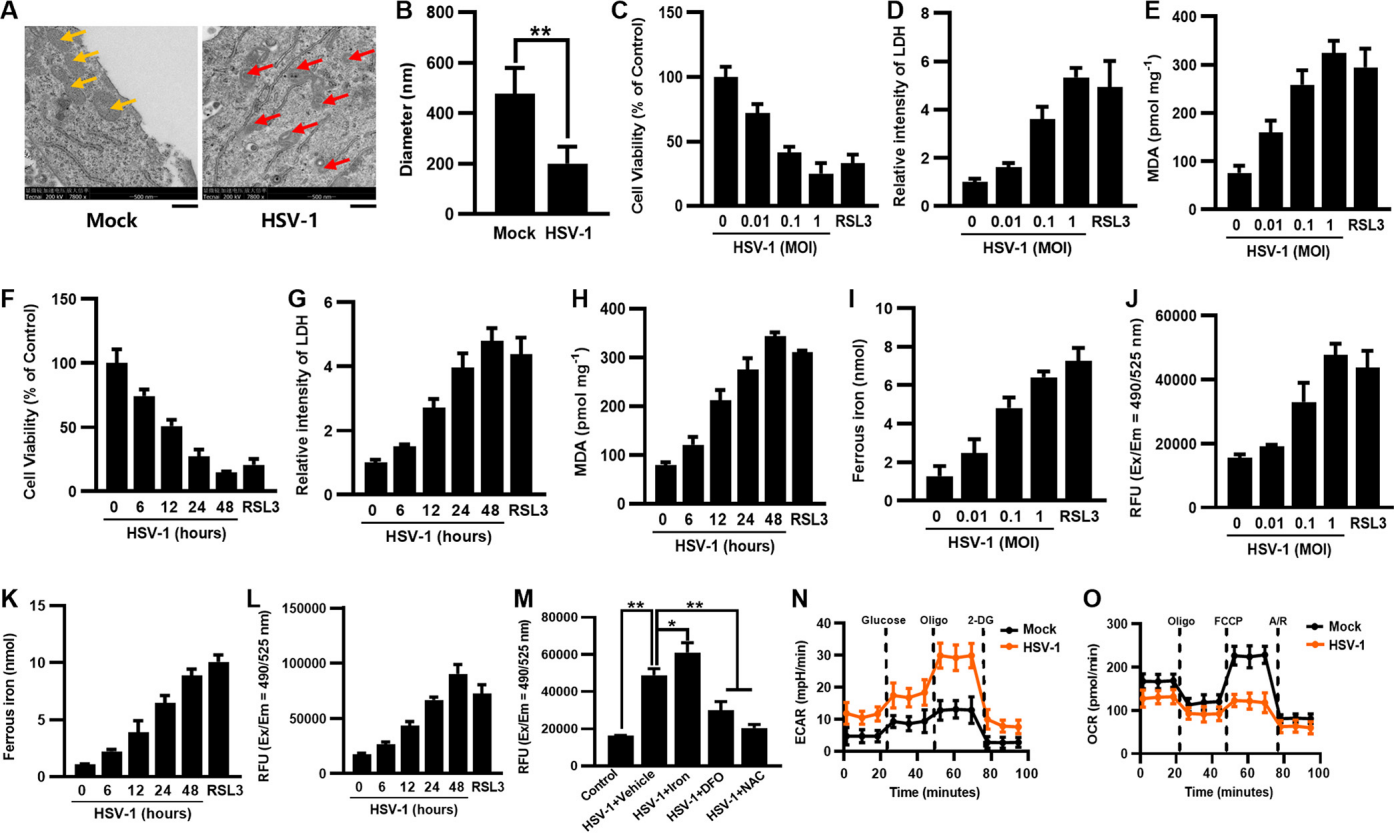
HSV-1 induces ferroptosis in U373 cells. (A) U373 cells were infected or not infected with (MOI = 0.1) for 24 h, and images were obtained by transmission electron microscopy at magnifications of ×7,800. The yellow arrows indicate normal mitochondria in mock-infected cells, and the red ones indicate shrunken mitochondria in HSV-1-infected cells. Representative images are shown. Bars, 500 nm. (B) The diameter of the mitochondria was quantified. **, *P < *0.01 (as measured by an unpaired *t* test). (C to H) U373 cells were infected with HSV-1 at an MOI of 0.01, 0.1, or 1 for 24 h (C to E) or an MOI of 0.1 for 6, 12, 24, or 48 h (F to H). Cells treated with RSL3 (5 μM) for 24 h were used as the positive control. (C and F) Cell viability was determined by a CCK8 assay, and the level of cell viability in uninfected cells was defined as 100%. (D and G) LDH released into the supernatants was determined by a cytotoxicity assay, and the level of LDH in uninfected cells was defined as 1-fold. (E and H) The MDA concentrations in cell lysates were determined by an MDA assay. (I and K) The ferrous iron concentrations in cell lysates were determined by an iron assay. (J and L) The ROS levels in cell lysates were determined by an ROS fluorometric assay. RFU, relative fluorescence units; Ex/Em, excitation/emission wavelength. (M) U373 cells were treated with exogenous iron (10 μM), deferoxamine (DFO) (100 μM), or *N*-acetylcysteine (NAC) (5 mM), followed by infection with HSV-1 (MOI = 0.1) for 24 h. The ROS levels in the cell lysates were determined by an ROS fluorometric assay. Data represent means ± SD from three repeated experiments. *, *P < *0.05; **, *P < *0.01 (as measured by two-way ANOVA). (N and O) U373 cells were infected with HSV-1 (MOI = 0.1) for 24 h and subjected to Seahorse analysis. Real-time changes in the ECAR (N) or OCR (O) of HSV-1-infected U373 cells after treatment with the indicated inhibitors are shown A/R, antimycin A and rotenone.

Astrocytes and microglia cells play critical roles in HSV-caused neurological disease in the CNS. Thus, to evaluate whether HSV-1 induces ferroptosis in these cells, we first examined the infection efficiency of HSV-1 in human astrocytoma U373 cells and microglial HMC3 cells. HSV-1 infection at a multiplicity of infection (MOI) of either 0.1 or 1 increased with increasing durations, as determined by measuring the mRNA level of HSV-1 glycoprotein D (gD), gD fluorescence staining, and observing the cytopathic effect (see Fig. S1A to D in the supplemental material). At 24 h postinfection (hpi), gD fluorescence was observed in the majority of cells infected with HSV-1 at an MOI of 0.1 (Fig. S1C and D), indicating a high infection efficiency. After that, we examined the morphological phenotype of mitochondria in U373 cells and HMC3 cells infected with HSV-1 at an MOI of 0.1 for 24 h since a typical morphological feature of ferroptosis is the shrinkage of mitochondria (20). Infected U373 and HMC3 cells displayed shrunken intracellular mitochondria compared with those of uninfected cells (Fig. 1A and B and Fig. S2A and B). Moreover, mitochondrion shrinkage in HSV-1-infected U373 and HMC3 cells was coincident with increases in cell death and lipid peroxidation (Fig. 1C to H and Fig. S2C to H), other characteristics of ferroptosis, which were examined by using cell counting kit 8 (CCK8), detecting the release of cytosolic lactate dehydrogenase (LDH), and determining the concentration of malondialdehyde (MDA) (an indicator of lipid peroxidation), respectively. In addition, cell death and lipid peroxidation were induced by HSV-1 infection in a dose- and time-dependent manner in both cell lines (Fig. 1C to H and Fig. S2C to H). The ferroptosis inducer RSL3 was used as a positive control.

10.1128/mbio.02370-22.1 FIG S1 Infection efficiency of HSV-1 in U373 and HMC3 cells. (A and B) U373 and HMC3 cells were infected with HSV-1 at an MOI of 0.1 or 1 for 6, 12, 24, and 48 h, and the cytopathic effect (CPE) was examined under an inverted microscope. Bars, 100 μm. (C) The expression levels of the HSV-1 gD genes in U373 cells infected with HSV-1 (MOI = 0.1 or 1) were determined by qRT-PCR at 6, 12, 24, or 48 hpi. The relative mRNA level of HSV-1 gD in uninfected cells was defined as 1-fold. Data represent means ± SD from three repeated experiments. **, *P < *0.01 (as measured by one-way ANOVA). (D) U373 cells were infected or not infected with HSV-1 (MOI = 0.1 or 1) for 6, 12, 24, or 48 h and then subjected to immunofluorescence staining with anti-HSV-1 gD antibody (green). DAPI was used for nuclear staining (blue). Representative images were acquired using fluorescence microscopy. Bar, 20 μm. Download FIG S1, TIF file, 0.3 MB. Copyright © 2022 Xu et al. 2022 Xu et al. https://creativecommons.org/licenses/by/4.0/ This content is distributed under the terms of the Creative Commons Attribution 4.0 International license.

10.1128/mbio.02370-22.2 FIG S2 HSV-1 induces ferroptosis in HMC3 cells. (A) HMC3 cells were infected or not infected with HSV-1 (MOI = 0.1) for 24 h, and images were obtained by transmission electron microscopy at magnifications of ×7,800. The yellow arrows indicate normal mitochondria in mock-infected cells, and the red ones indicate shrunken mitochondria in HSV-1-infected cells. Representative images are shown. Bar, 500 nm. (B) The diameter of the mitochondria was quantified. **, *P < *0.01 (as measured by an unpaired *t* test). (C to H) HMC3 cells were infected with HSV-1 at an MOI of 0.01, 0.1, or 1 for 24 h (C to E) or an MOI of 0.1 for 6, 12, 24, or 48 h (F to H). Cells treated with RSL3 (5 μM) for 24 h were used as the positive control. (C and F) Cell viability was determined by a CCK8 assay, and the level of cell viability in uninfected cells was defined as 100%. (D and G) The LDH released into the supernatants was determined by a cytotoxicity assay, and the level of LDH in uninfected cells was defined as 1-fold. (E and H) The MDA concentrations in cell lysates were determined by an MDA assay. (I and K) The ferrous iron concentrations in cell lysates were determined by an iron assay. (J and L) The ROS levels in the cell lysates were determined by an ROS fluorometric assay. (M) HMC3 cells were treated with exogenous iron (10 μM), DFO (100 μM), or NAC (5 mM), followed by infection with HSV-1 (MOI = 0.1) for 24 h. The ROS level in cell lysates was determined by an ROS fluorometric assay. Data represent means ± SD from three repeated experiments. **, *P < *0.01 (as measured by two-way ANOVA). (N and O) HMC3 cells were infected with HSV-1 (MOI = 0.1) for 24 h and subjected to Seahorse analysis. Real-time changes in the ECAR (N) or OCR (O) of HSV-1-infected HMC3 cells after treatment with the indicated inhibitors are shown. Download FIG S2, TIF file, 0.1 MB. Copyright © 2022 Xu et al. 2022 Xu et al. https://creativecommons.org/licenses/by/4.0/ This content is distributed under the terms of the Creative Commons Attribution 4.0 International license.

Lipid peroxidation results from the iron-dependent production of excessive ROS (5). Thus, we measured the intracellular levels of Fe^2+^ and ROS in HSV-1-infected cells using an iron assay kit and a fluorometric intracellular ROS kit, respectively. Our results showed that HSV-1 induced the accumulation of Fe^2+^ and ROS in a dose- and time-dependent manner (Fig. 1I to L and Fig. S2I to L). Moreover, the addition of exogenous iron further increased the ROS levels in HSV-1-infected U373 and HMC3 cells, whereas treatment with an iron chelator (deferoxamine [DFO]) or an antioxidant (*N*-acetylcysteine [NAC]) significantly reduced the ROS levels (Fig. 1M and Fig. S2M). These results indicate that HSV-1-induced ROS accumulation is iron dependent. Moreover, because the production of ROS is tightly related to glycolysis and the tricarboxylic acid (TCA) cycle (21, 22), we further examined glycolysis and oxidative phosphorylation (OXPHOS) in HSV-1-infected cells by measuring the extracellular acidification rate (ECAR) and the cellular oxygen consumption rate (OCR). Seahorse analysis showed that HSV-1 increased the glycolytic capacity (Fig. 1N and Fig. S2N) and reduced the spare respiratory capacity (Fig. 1O and Fig. S2O) in U373 and HMC3 cells, consistent with previous observations that a reduction in respiration induced by pathogen infection or lipopolysaccharide (LPS) treatment can lead to an increase in ROS production (23).

**FIG 2:**
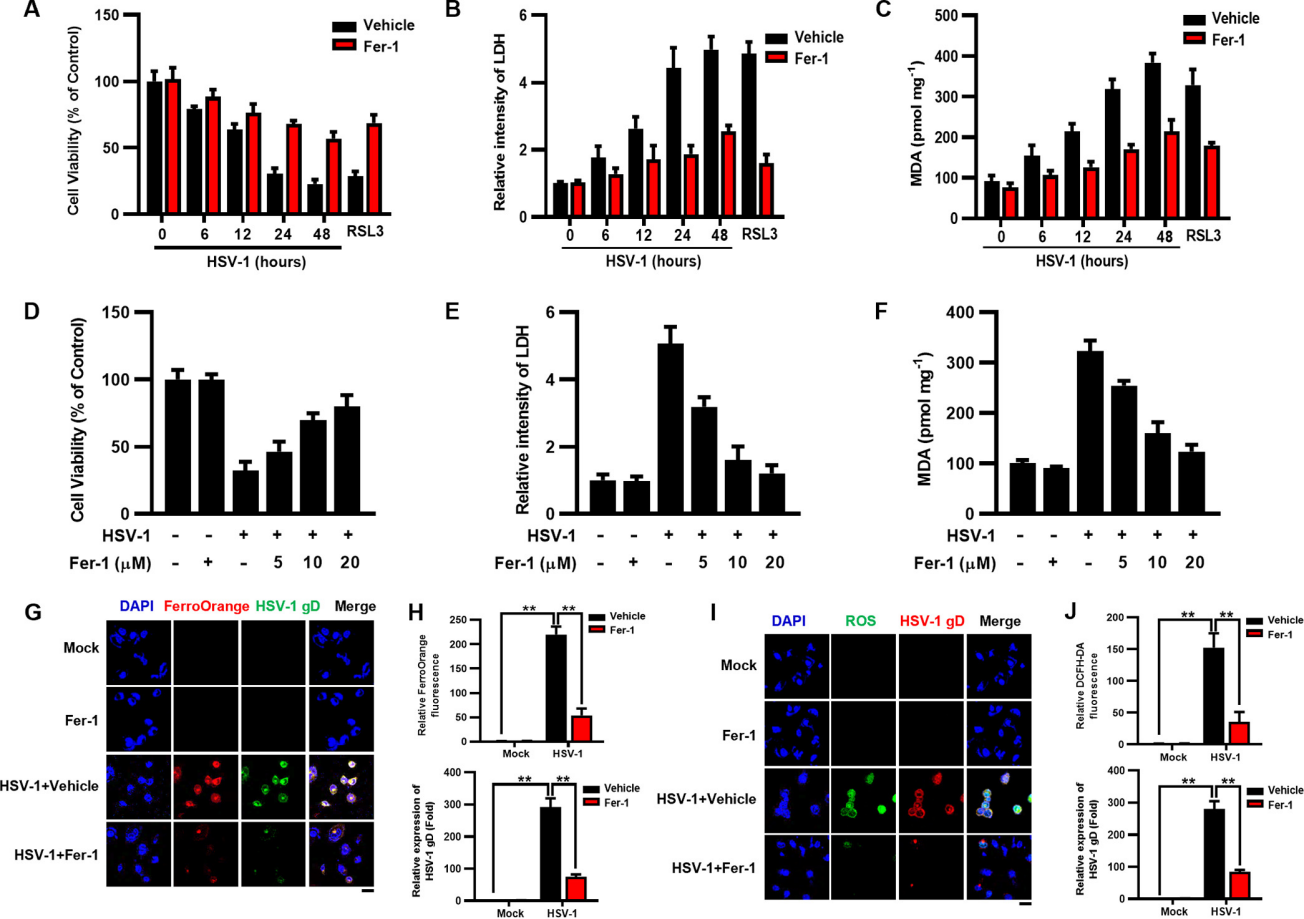
Fer-1 inhibits HSV-1-induced ferroptosis in U373 cells. (A to F) U373 cells were treated with Fer-1 (10 μM) or the vehicle (DMSO), followed by infection with HSV-1 (MOI = 0.1) for 6, 12, 24, or 48 h (A to C), or U373 cells were treated with Fer-1 (5, 10, or 20 μM) or the vehicle (DMSO), followed by infection with HSV-1 (MOI = 0.1) for 24 h (D to F). (A and D) Cell viability was determined by a CCK8 assay, and the level of cell viability in uninfected cells treated with the vehicle was defined as 100%. (B and E) LDH released into the supernatants was determined by a cytotoxicity assay, and the level of LDH in uninfected cells treated with the vehicle was defined as 1-fold. (C and F) The MDA concentrations in cell lysates were determined by an MDA assay. (G to J) U373 cells were infected with HSV-1 (MOI = 0.1), treated with or without Fer-1 for 24 h, and then subjected to immunofluorescence staining. The levels of Fe^2+^ (G and H) and ROS (I and J) were determined using FerroOrange (red) and DCFH-DA (green) probes, respectively. The levels of HSV-1 infection were determined using anti-HSV-1 gD antibody. DAPI was used for nuclear staining (blue). Bars, 20 μm. The relative fluorescence intensities of FerroOrange, DCFH-DA, and HSV-1 gD were quantified using ImageJ software, and the level of fluorescence signals in uninfected cells treated with the vehicle was defined as 1-fold (H and J). Representative images were acquired using fluorescence microscopy. **, *P < *0.01 (as measured by two-way ANOVA).

We sought to examine whether the inhibition of ferroptosis can protect against HSV-1-induced cell death and lipid peroxidation. U373 and HMC3 cells were treated with ferrostatin-1 (Fer-1) (10 μM) or a vehicle (dimethyl sulfoxide [DMSO]), followed by infection with HSV-1 (MOI = 0.1). Fer-1 is a potent ferroptosis inhibitor that has been reported to prevent iron overload and ROS accumulation (20). Our results showed that Fer-1 treatment enhanced cell viability (Fig. 2A and B and Fig. S3A and B) and inhibited lipid peroxidation (Fig. 2C and Fig. S3C) in HSV-1-infected cells during the infection process. Moreover, the rescuing effects of Fer-1 on HSV-1-induced cell death and lipid peroxidation were also dose dependent (Fig. 2D to F and Fig. S3D to F). In addition, we examined the intracellular levels of iron and ROS in HSV-1-infected cells treated with or without Fer-1 using confocal microscopy. An iron probe (FerroOrange) and a dichlorofluorescein diacetate (DCFH-DA) probe were used to detect the signals of Fe^2+^ and ROS, respectively. As a result, HSV-1 significantly induced the accumulation of Fe^2+^ and ROS, while Fer-1 treatment remarkably ameliorated such inductions in HSV-1-infected U373 and HMC3 cells (Fig. 2G to J and Fig. S3G to J). Moreover, the Fe^2+^ and ROS signals tightly colocalized with the HSV-1 gD protein, confirming that the increased intracellular ROS/iron levels were attributed to infection with HSV-1.

10.1128/mbio.02370-22.3 FIG S3 Fer-1 inhibits HSV-1-induced ferroptosis in HMC3 cells. (A to F) HMC3 cells were treated with Fer-1 (10 μM) or the vehicle (DMSO), followed by infection with HSV-1 (MOI = 0.1) for 6, 12, 24, or 48 h (A to C), or HMC3 cells were treated with Fer-1 (5, 10, or 20 μM) or the vehicle (DMSO), followed by infection with HSV-1 (MOI = 0.1) for 24 h (D to F). (A and D) Cell viability was determined by a CCK8 assay, and the level of cell viability in uninfected cells treated with the vehicle was defined as 100%. (B and E) The LDH released into the supernatants was determined by a cytotoxicity assay, and the level of LDH in uninfected cells treated with the vehicle was defined as 1-fold. (C and F) The MDA concentrations in cell lysates were determined by an MDA assay. (G to J) HMC3 cells were infected with HSV-1 (MOI = 0.1), treated with or without Fer-1 for 24 h, and then subjected to immunofluorescence staining. The levels of Fe^2+^ (G and H) and ROS (I and J) were determined using the FerroOrange (red) and DCFH-DA (green) probes, respectively. The levels of HSV-1 infection were determined using anti-HSV-1 gD antibody. DAPI was used for nuclear staining (blue). Bar, 20 μm. The relative fluorescence intensities of FerroOrange, DCFH-DA, and HSV-1 gD were quantified using ImageJ software, and the level of fluorescence signals in uninfected cells treated with the vehicle was defined as 1-fold (H and J). Representative images were acquired using fluorescence microscopy. **, *P < *0.01 (as measured by two-way ANOVA). Download FIG S3, TIF file, 0.1 MB. Copyright © 2022 Xu et al. 2022 Xu et al. https://creativecommons.org/licenses/by/4.0/ This content is distributed under the terms of the Creative Commons Attribution 4.0 International license.

Interestingly, we observed that Fer-1 treatment significantly suppressed HSV-1 replication in both U373 and HMC3 cells (Fig. S4A to D). Previous studies have shown that HSV-1 induces the production of ROS in murine neural cells (17, 18). Moreover, the inhibition of ROS accumulation by antioxidants can suppress HSV-1 replication (19, 24). Thus, we speculate that intracellular ROS accumulation is important for HSV-1 replication. To test this possibility, we examined intracellular ROS levels and HSV-1 replication in infected cells treated with exogenous ROS in the presence and absence of Fer-1, respectively. The addition of exogenous ROS (H_2_O_2_) significantly enhanced intracellular ROS levels and HSV-1 gD expression in HSV-1-infected cells, whereas Fer-1 treatment reversed such enhancements (Fig. S4E to H), indicating that Fer-1 can inhibit HSV-1 replication by preventing virus-induced ROS accumulation.

10.1128/mbio.02370-22.4 FIG S4 Fer-1 treatment inhibits HSV-1 replication in U373 and HMC3 cells. (A to D) U373 (A and B) and HMC3 (C and D) cells were treated with Fer-1 (10 μM) or the vehicle (DMSO), followed by infection with HSV-1 (MOI = 0.1) for 24 h. (A and C) Total RNAs were extracted and subjected to qPCR to assess viral mRNA accumulation, and the mRNA level of HSV-1 gD in infected cells treated with the vehicle was defined as 100%. (B and D) The viral titer in the supernatants was determined by a plaque assay. *, *P < *0.05; **, *P < *0.01 (as measured by an unpaired *t* test). (E to H) U373 (E and F) and HMC3 (G and H) cells were treated with Fer-1 (10 μM) and/or H_2_O_2_ (1 mM), followed by infection with HSV-1 (MOI = 0.1) for 24 h. (E and G) The ROS levels in the cell lysates were determined by an ROS fluorometric assay. (F and H) Viral mRNA accumulation was determined by qRT-PCR, and the mRNA level of HSV-1 gD in infected cells treated with the vehicle was defined as 100%. Data represent means ± SD from three repeated experiments. *, *P < *0.05; **, *P < *0.01 (as measured by two-way ANOVA). Download FIG S4, TIF file, 0.1 MB. Copyright © 2022 Xu et al. 2022 Xu et al. https://creativecommons.org/licenses/by/4.0/ This content is distributed under the terms of the Creative Commons Attribution 4.0 International license.

Subsequently, we sought to examine the relationship between ferroptosis and other types of cell death, including apoptosis and necroptosis. Flow cytometry with annexin V-allophycocyanin (fluorescein isothiocyanate [FITC])–propidium iodide (PI) double staining was used to detect apoptosis, and Western blotting with antibodies against phosphorylated mixed-lineage kinase domain-like protein (p-MLKL) and phosphorylated receptor-interacting serine/threonine protein kinase 3 (p-RIPK3) was used to detect necroptosis signaling. Staurosporine (STS) (an apoptosis inducer) and tumor necrosis factor alpha (TNF-α) (a necroptosis inducer) were used as positive controls. Annexin V and PI staining was significantly increased in HSV-1-infected U373 cells compared to that in mock-infected cells, indicating that HSV-1 induced apoptotic cell death (Fig. S5A and B). Besides, HSV-1 resulted in the upregulated expression of the necroptosis markers p-MLKL and p-RIPK3 (Fig. S5C). These results uncovered that HSV-1 also induced other types of programmed cell death in U373 cells. Moreover, Fer-1 treatment showed no or little effect on the apoptosis rate or necroptosis signaling in HSV-1-infected cells (Fig. S5A to C), confirming that the rescuing effects of Fer-1 on HSV-1 infection were via inhibiting ferroptosis.

10.1128/mbio.02370-22.5 FIG S5 Fer-1 treatment showed no or little effect on the apoptosis rate or necroptosis signaling in HSV-1-infected cells. (A and B) U373 cells were treated with Fer-1 (10 μM) or the vehicle (DMSO), followed by infection with HSV-1 (MOI = 0.1) for 24 h. STS (apoptosis inducer) (1 μM) was used as a positive control. Cells were collected and stained with annexin V-FITC–PI for flow cytometry analysis, and the percentage of apoptotic cells was measured. Data represent means ± SD. **, *P < *0.01; n.s., no significance (as measured by two-way ANOVA). (C) Total proteins were subjected to Western blotting with antibodies to MLKL, p-MLKL, RIPK3, p-RIPK3, and GAPDH. TNF-α (necroptosis inducer) (100 ng/mL) was used as a positive control. Download FIG S5, TIF file, 0.1 MB. Copyright © 2022 Xu et al. 2022 Xu et al. https://creativecommons.org/licenses/by/4.0/ This content is distributed under the terms of the Creative Commons Attribution 4.0 International license.

Together, our findings indicate that HSV-1 induces ferroptosis in cultured human astrocytes and microglia cells.

### HSV-1 interrupts the biosynthesis of GSH in human astrocytes and microglia cells.

**FIG 3:**
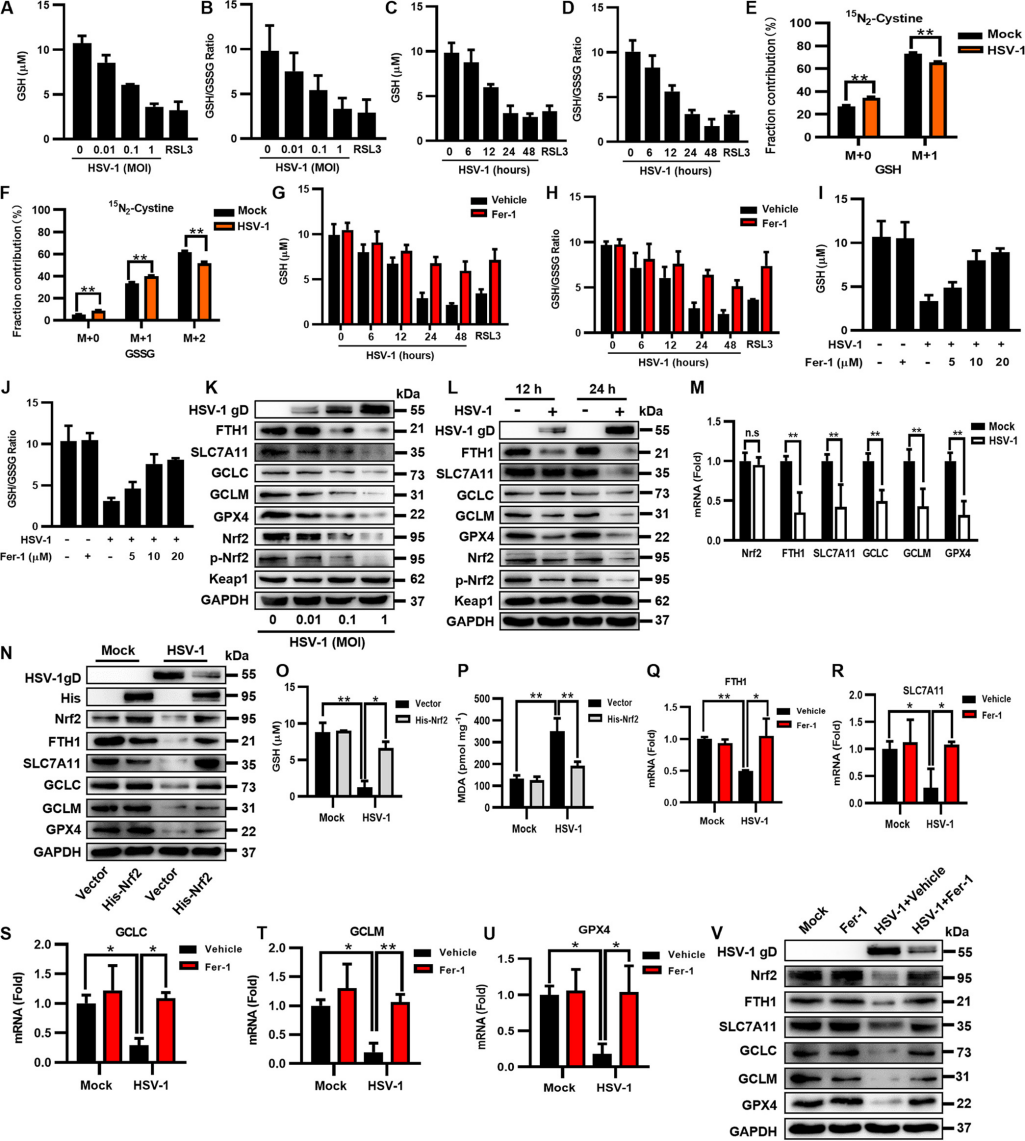
HSV-1 interrupts the biosynthesis of GSH by downregulating the protein level of Nrf2 in U373 cells. (A to D) U373 cells were infected with HSV-1 at an MOI of 0.01, 0.1, or 1 for 24 h (A and B) or an MOI of 0.1 for 6, 12, 24, or 48 h (C and D). The intracellular GSH level and the GSH/GSSG ratio were measured using a GSSG/GSH quantification kit. (E and F) U373 cells were incubated in cystine-free medium supplemented with 0.26 mM [^15^N_2_]cystine and then infected with HSV-1 (MOI = 0.1) for 12 h. The extracted metabolites were analyzed by LC-MS/MS, and the distributions of isotopically labeled GSH and GSSG are presented. Data represent means ± SD from three repeated experiments. **, *P < *0.01 (as measured by two-way ANOVA). (G to J) U373 cells were treated with Fer-1 (10 μM) or the vehicle (DMSO), followed by infection with HSV-1 (MOI = 0.1) for 6, 12, 24, or 48 h (G and H), or U373 cells were treated with Fer-1 (5, 10, or 20 μM) or the vehicle (DMSO), followed by infection with HSV-1 (MOI = 0.1) for 24 h (I and J). The intracellular GSH level and the GSH/GSSG ratio were measured using a GSSG/GSH quantification kit. (K and L) U373 cells were infected with HSV-1 at an MOI of 0.01, 0.1, or 1 for 24 h (K) or an MOI of 0.1 for 12 or 24 h (L). Total proteins were subjected to Western blotting with antibodies to HSV-1 gD, FTH1, SLC7A11, GCLC, GCLM, GPX4, Nrf2, p-Nrf2, Keap1, and GAPDH. (M) The expression levels of the indicated genes in U373 cells infected with HSV-1 (MOI = 0.1) were determined by qRT-PCR at 24 hpi. The relative mRNA level of each gene in uninfected cells was defined as 1-fold. Data represent means ± SD from three repeated experiments. **, *P < *0.01; n.s, no significance (as measured by an unpaired *t* test). (N to P) U373 cells were transfected with a plasmid encoding His-Nrf2 or an empty vector and then infected with HSV-1 (MOI = 0.1). (N) At 24 hpi, total proteins were subjected to Western blotting with the indicated antibodies. (O) The intracellular GSH level was measured using a GSSG/GSH quantification kit. (P) The intracellular MDA concentration was determined by an MDA assay. Data represent means ± SD from three repeated experiments. *, *P < *0.05; **, *P < *0.01 (as measured by two-way ANOVA). (Q to V) U373 cells were treated with Fer-1 (10 μM) or the vehicle (DMSO), followed by infection with HSV-1 (MOI = 0.1) for 24 h. (Q to U) Total RNAs were subjected to qRT-PCR to determine the expression levels of the indicated genes, and the relative mRNA level of each indicated gene in uninfected cells treated with the vehicle was defined as 1-fold. Data represent means ± SD from three repeated experiments. *, *P < *0.05; **, *P < *0.01 (as measured by two-way ANOVA). (V) Total proteins were subjected to Western blotting with the indicated antibodies.

The accumulation of excessive ROS can lead to lipid peroxidation and ferroptosis (25). In response, intracellular GSH is oxidized to GSSG to scavenge massive ROS under oxidative stress (7). Therefore, after identifying that HSV-1 induced excessive ROS production and ferroptosis in human neural cells, we sought to examine the GSH level and the GSH/GSSG ratio in HSV-1-infected cells using a GSSG/GSH quantification kit. Our data showed that both the GSH level and the GSH/GSSG ratio were decreased by HSV-1 in a dose- and time-dependent manner in both U373 and HMC3 cells (Fig. 3A to D and Fig. S6A to D), coinciding with the increases in ROS production, lipid peroxidation, and cell death (Fig. 1 and 2 and Fig. S2 and S3).

10.1128/mbio.02370-22.6 FIG S6 HSV-1 interrupts the biosynthesis of GSH in HMC3 cells. (A to D) HMC3 cells were infected with HSV-1 at an MOI of 0.01, 0.1, or 1 for 24 h (A and B) or an MOI of 0.1 for 6, 12, 24, or 48 h (C and D). The intracellular GSH level and the GSH/GSSG ratio were measured using a GSSG/GSH quantification kit. (E to H) HMC3 cells were treated with Fer-1 (10 μM) or the vehicle (DMSO), followed by infection with HSV-1 (MOI = 0.1) for 6, 12, 24, or 48 h (E and F), or HMC3 cells were treated with Fer-1 (5, 10, or 20 μM) or the vehicle (DMSO), followed by infection with HSV-1 (MOI = 0.1) for 24 h (G and H). The intracellular GSH level and the GSH/GSSG ratio were measured using a GSSG/GSH quantification kit. Download FIG S6, TIF file, 0.1 MB. Copyright © 2022 Xu et al. 2022 Xu et al. https://creativecommons.org/licenses/by/4.0/ This content is distributed under the terms of the Creative Commons Attribution 4.0 International license.

Extracellular cystine is the primary source of intracellular cysteine, which is the synthetic substrate of GSH (26). To confirm that HSV-1 infection indeed decreased the intracellular GSH level, U373 cells were incubated in cystine-free medium supplemented with stable-isotope-labeled cystine ([^15^N_2_]cystine) and then infected with HSV-1 (MOI = 0.1) for 12 h to isotopically monitor the biosynthesis of GSH and GSSG. We observed that the labeling of GSH (M1 isotopologue [one isotope-labeled nitrogen]) and GSSG (M2 isotopologue) derived from the [^15^N_2_]cystine-carbon was significantly decreased in HSV-1-infected cells (Fig. 3E and F), consistent with the data obtained using the GSSG/GSH quantification kit (Fig. 3A to D).

We sought to examine the effect of inhibiting ferroptosis on HSV-1-induced GSH depletion. Thus, U373 and HMC3 cells were treated with Fer-1 (10 μM) or the vehicle (DMSO), followed by infection with HSV-1 (MOI = 0.1) for 24 h. Fer-1 treatment significantly increased the GSH level and the GSH/GSSG ratio in the infected cells (Fig. 3G to J and Fig. S6E to H) in a time- and dose-independent manner, suggesting that intracellular GSH is essential for scavenging HSV-1-induced ROS and the corresponding ferroptosis. To confirm this, U373 cells were infected with HSV-1 (MOI = 0.1) for 2 h and then treated with increasing concentrations of exogenous GSH (from 10 mM to 30 mM). Exogenous GSH supplementation increased the intracellular GSH level and the GSH/GSSG ratio in a dose-dependent manner in the infected cells at 24 hpi (Fig. S7A and B). In agreement with this, the intracellular level of ROS was substantially reduced when infected cells were supplemented with GSH (20 mM) (Fig. S7C), indicating that exogenous GSH neutralized the accumulation of excessive ROS induced by HSV-1. Besides, HSV-1 replication was also inhibited in the presence of exogenous GSH (Fig. S7D and E).

10.1128/mbio.02370-22.7 FIG S7 Exogenous GSH supplementation inhibits HSV-1 replication in U373 cells. (A to C) U373 cells were infected with HSV-1 (MOI = 0.1) for 2 h and then treated with the indicated concentrations of GSH for another 24 h. (A and B) The intracellular GSH level and the GSH/GSSG ratio were measured using a GSSG/GSH quantification kit. (C) The ROS level in cell lysates was determined using an ROS fluorometric assay. Data represent means ± SD from three repeated experiments. **, *P < *0.01 (as measured by two-way ANOVA). (D) U373 cells were infected with HSV-1 (MOI = 0.1) for 2 h and then treated with the indicated concentrations of GSH for another 24 h. Total RNAs were extracted and subjected to qPCR to assess viral RNA accumulation, and the RNA level of HSV-1 gD in infected cells treated with the vehicle was defined as 100%. (E) The viral titer in the supernatants was determined by a plaque assay. Data represent means ± SD from three repeated experiments. *, *P < *0.05; **, *P < *0.01 (as measured by two-way ANOVA). Download FIG S7, TIF file, 0.1 MB. Copyright © 2022 Xu et al. 2022 Xu et al. https://creativecommons.org/licenses/by/4.0/ This content is distributed under the terms of the Creative Commons Attribution 4.0 International license.

Based on these results, we conclude that the biosynthesis of GSH is interrupted in HSV-1-infected astrocytes and microglia cells, and this process contributes to excessive ROS accumulation and ferroptosis.

### HSV-1 interrupts the biosynthesis of GSH by downregulating the protein level of Nrf2.

To further explore the details of HSV-1-induced ferroptosis, we focused on the antioxidative genes implicated in negatively regulating ferroptosis, including ferritin heavy chain 1 (FTH1), cystine/glutamate antiporter xCT (SLC7A11), the glutamate-cysteine ligase catalytic subunit (GCLC), the glutamate-cysteine ligase modifier (GCLM), and GPX4. FTH1 is a major component of ferritin, an iron storage protein complex that prevents the iron-mediated production of ROS (27). SLC7A11 is a key component of the Xc system that ingests extracellular cystine in cells, which is then reduced to cysteine for GSH biosynthesis (28). The glutamate cysteine ligase, composed of GCLC and GCLM, catalyzes the rate-limiting step of GSH biosynthesis (29). GPX4 mediates the process of GSH oxidization to GSSG for scavenging excessive ROS (30). Western blotting showed that the protein levels of these genes were reduced in response to HSV-1 in a dose- and time-dependent manner in both U373 and HMC3 cells (Fig. 3K and L and Fig. S8A and B), consistent with the results from HSV-1-induced ROS production/GSH depletion and ferroptosis. Moreover, the mRNA levels of these genes were also significantly reduced in HSV-1-infected cells compared to those in uninfected cells (Fig. 3M and Fig. S8C).

10.1128/mbio.02370-22.8 FIG S8 HSV-1 interrupts the biosynthesis of GSH by downregulating the protein level of Nrf2 in HMC3 cells. (A and B) HMC3 cells were infected with HSV-1 at an MOI of 0.01, 0.1, or 1 for 24 h (A) or an MOI of 0.1 for 12 or 24 h (B). Total proteins were subjected to Western blotting with antibodies. (C) The expression levels of the indicated genes in HSV-1-infected HMC3 cells were determined by qRT-PCR at 24 hpi. The relative mRNA level of each indicated gene in uninfected cells was defined as 1-fold. Data represent means ± SD from three repeated experiments. *, *P < *0.05; **, *P < *0.01; n.s., no significance (as measured by an unpaired *t* test). (D to I) HMC3 cells were treated with Fer-1 (10 μM) or the vehicle (DMSO), followed by infection with HSV-1 (MOI = 0.1) for 24 h. (D) Total proteins were subjected to Western blotting with the indicated antibodies. (E to I) Total RNAs were subjected to qRT-PCR to determine the expression levels of the indicated genes, and the relative mRNA level of each indicated gene in uninfected cells treated with the vehicle was defined as 1-fold. Data represent means ± SD from three repeated experiments. *, *P < *0.05; **, *P < *0.01 (as measured by two-way ANOVA). Download FIG S8, TIF file, 0.1 MB. Copyright © 2022 Xu et al. 2022 Xu et al. https://creativecommons.org/licenses/by/4.0/ This content is distributed under the terms of the Creative Commons Attribution 4.0 International license.

The simultaneous downregulation of the expression levels of FTH1, SLC7A11, GCLC, GCLM, and GPX4 in HSV-1-infected cells suggests that the transcription factor Nrf2, responsible for the transcription of these genes, may be dysregulated during HSV-1 infection. Thus, we examined the expression level of Nrf2 in U373 cells and HMC3 cells infected with HSV-1. We found that the protein levels of Nrf2 and its active phosphorylated form (p-Nrf2) were dose- and time-dependently inhibited by HSV-1 (Fig. 3K and L and Fig. S8A and B, bottom), while the mRNA level of Nrf2 was not or only slightly affected (Fig. 3M and Fig. S8C), showing that HSV-1 regulates Nrf2 at the protein level. Of note, the protein level of Keap1, the interactor and repressor of Nrf2, was stable in response to HSV-1 (Fig. 3K and L, bottom), indicating that HSV-1 specifically targets Nrf2.

To confirm the role of Nrf2 in HSV-1-induced GSH depletion and ferroptosis, U373 cells were transfected with a plasmid encoding His-Nrf2 or an empty vector and then infected with HSV-1 for 24 h. The protein level of endogenous Nrf2 was remarkably reduced in the infected cells compared with that in uninfected cells, while the overexpression of His-Nrf2 counterbalanced the inhibitory effect of HSV-1 infection on the protein level of endogenous Nrf2 as well as the levels of its downstream genes (Fig. 3N). Moreover, the GSH level was significantly increased while the MDA level was significantly reduced in infected His-Nrf2-expressing cells compared with those in infected cells expressing the empty vector (Fig. 3O and P). Our findings indicate that overexpressing Nrf2 reversed HSV-1-induced GSH depletion, thereby ameliorating the process of ferroptosis. In addition, we examined ferroptosis-related genes in HSV-1-infected cells treated with or without Fer-1. As a result, the reductions in the protein and mRNA levels of these genes were reversed when infected cells were treated with Fer-1 (Fig. 3Q to V and Fig. S8D to I), indicating feedback between ferroptosis and Nrf2 signaling during HSV-1 infection. Together, these results uncover that the downregulation of Nrf2 plays an important role in inducing GSH depletion and ferroptosis in HSV-1-infected cells.

Altogether, we conclude that HSV-1-induced Nrf2 downregulation leads to the loss of its antiferroptotic activity, which contributes to interrupting GSH biosynthesis and promoting ferroptosis.

### HSV-1 inhibits Nrf2 by accelerating its ubiquitination and degradation dependent on Keap1.

**FIG 4:**
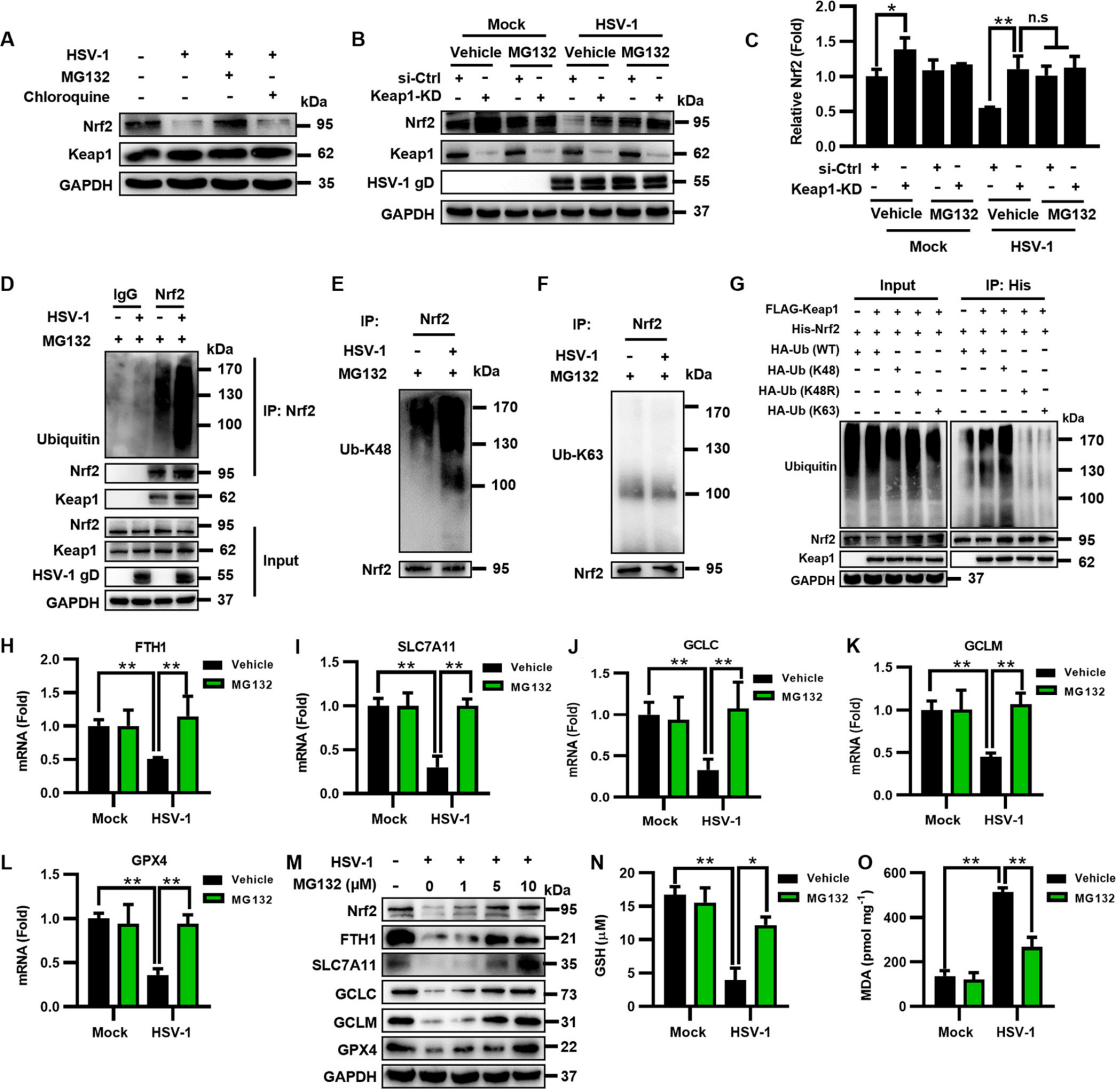
HSV-1 inhibits Nrf2 by accelerating its ubiquitination and degradation dependent on Keap1. (A) U373 cells were infected with HSV-1 (MOI = 0.1) and then treated with the vehicle (DMSO), MG132 (10 μM), or chloroquine (10 μM). At 24 hpi, total proteins were extracted and subjected to Western blotting with the indicated antibodies. (B and C) U373 cells were transfected with siRNA targeting Keap1 (Keap1-KD) or control siRNA (si-Ctrl) for 24 h and then infected with HSV-1 (MOI = 0.1) in the presence or absence of MG132 (10 μM). (B) At 24 hpi, total proteins were subjected to Western blotting with the indicated antibodies. (C) Relative quantification of Nrf2 normalized by GAPDH was performed using ImageJ software, and the relative protein level of Nrf2 in uninfected si-Ctrl cells treated with the vehicle was defined as 1-fold. *, *P < *0.05; **, *P < *0.01; n.s, no significance (as measured by two-way ANOVA). (D) U373 cells were infected with HSV-1 (MOI = 0.1) in the presence or absence of MG132 (10 μM) for 24 h and subjected to coimmunoprecipitation with anti-Nrf2 or anti-IgG antibody, followed by immunoblotting with the indicated antibodies. (E and F) The Nrf2-bound precipitates were analyzed using anti-Ub_K48_ and anti-Ub_K63_ antibodies. (G) HEK293T cells were cotransfected with FLAG-Keap1 and His-Nrf2 together with HA-Ub_WT_, HA-Ub_K48_, HA-Ub_K48R_, or HA-Ub_K63_ for 24 h and subjected to coimmunoprecipitation with anti-His antibody, followed by immunoblotting with the indicated antibodies. An empty vector was used to make sure that the total amounts of plasmids used in each sample were equal. (H to L) U373 cells were treated with MG132 (10 μM) or the vehicle (DMSO), followed by infection with HSV-1 (MOI = 0.1) for 24 h. Total RNAs were subjected to qRT-PCR to determine the expression levels of the indicated genes, and the relative mRNA level of each indicated gene in uninfected cells treated with the vehicle was defined as 1-fold. Data represent means ± SD from three repeated experiments. **, *P < *0.01 (as measured by two-way ANOVA). (M) U373 cells were infected with HSV-1 (MOI = 0.1) and then treated with MG132 at a concentration of 1, 5, or 10 μM. At 24 hpi, total proteins were subjected to Western blotting with the indicated antibodies. (N and O) U373 cells were treated with MG132 (10 μM) or the vehicle (DMSO), followed by infection with HSV-1 (MOI = 0.1) for 24 h. (N) The intracellular GSH level was measured using a GSSG/GSH quantification kit. (O) The intracellular MDA concentration was determined using an MDA assay.

Previous studies have shown that HSV-1 can hijack ubiquitin (Ub) machinery to establish a supportive microenvironment for efficient replication (31). Because HSV-1 targets Nrf2 at its protein level, we speculate that HSV-1 may induce the downregulation of Nrf2 via the UPS. To test this possibility, we examined the Nrf2 protein level in HSV-1-infected U373 cells in the presence of MG132 (a proteasome inhibitor) or chloroquine (an autophagosome-lysosome inhibitor for a control). Our results showed that HSV-1 infection substantially reduced the protein level of Nrf2 in vehicle (DMSO)-treated cells, while the Nrf2 protein level was stable in cells treated with MG132 (Fig. 4A). In contrast, chloroquine treatment did not restore the Nrf2 protein level in the infected cells (Fig. 4A), showing that HSV-1-induced Nrf2 degradation was not dependent on autophagy.

The E3 ubiquitin ligase Keap1 regulates the protein level of Nrf2 via the UPS (9). To test whether HSV-1-induced Nrf2 degradation depends on Keap1, we knocked down Keap1 via RNA interference (RNAi) in U373 cells infected or not infected with HSV-1 (MOI = 0.1) and then examined the protein level of Nrf2 in the presence or absence of MG132 at 24 hpi. As shown in Fig. 4B and C, Keap1 deficiency indeed enhanced the protein level of Nrf2 in uninfected or infected DMSO-treated cells, while MG132 treatment inhibited such enhancements in Keap1-KD (Keap1 knockdown) cells. Our findings indicate that Keap1 is required for HSV-1-induced Nrf2 degradation.

To determine the details of Keap1 regulating Nrf2 during HSV-1 infection, U373 cells were infected with HSV-1 (MOI = 0.1) in the presence of MG132 and then subjected to coimmunoprecipitation (co-IP) with anti-Nrf2 antibody at 24 hpi, followed by immunoblotting with anti-Ub and anti-Keap1 antibodies to detect the Keap1-Nrf2 interaction and ubiquitinated Nrf2. Interestingly, we observed that HSV-1 infection enhanced the ubiquitination of Nrf2 (Fig. 4D). Moreover, examining the Nrf2-bound immunoprecipitants with anti-Ub-Lys48 (K48) and anti-Ub_K63_ antibodies showed that HSV-1 infection promoted the formation of K48-linked polyubiquitin chains on Nrf2 (Fig. 4E and F). To confirm this, HEK293T cells were cotransfected with FLAG-Keap1 and His-Nrf2, together with hemagglutinin (HA)-tagged wild-type Ub (Ub_WT_), HA-Ub_K48_, HA-Ub_K48R_ (a K48 Ub mutant that results in the premature termination of ubiquitin chains), or HA-Ub_K63_. We observed that the overexpression of Ub_K48_ efficiently induced Nrf2 ubiquitination, whereas Ub_K48R_ and Ub_K63_ were unable to produce K48 or K63 polyubiquitin chains (Fig. 4G). Our findings indicate that HSV-1 infection enhances Keap1-dependent Nrf2 degradation via K48-linked polyubiquitin.

To confirm the effect of HSV-1-enhanced Nrf2 degradation on ferroptosis, we examined the expression levels of antiferroptotic genes as well as the intracellular levels of GSH and ROS in HSV-1-infected U373 cells in the presence or absence of MG132. The mRNA and protein levels of the genes downstream of Nrf2, including FTH1, SLC7A11, GCLC, GCLM, and GPX4, in MG132-treated infected cells were significantly increased compared with those in DMSO-treated infected cells (Fig. 4H to M). Moreover, MG132 treatment increased the GSH level and inhibited lipid peroxidation in HSV-1-infected cells (Fig. 4N and O), coinciding with the increased protein levels of Nrf2 as well as its downstream antiferroptotic genes.

Altogether, we conclude that HSV-1 infection can enhance Keap1-dependent Nrf2 degradation, thereby disrupting cellular redox homeostasis and promoting ferroptosis.

### HSV-1 induces ferroptosis in murine brain astrocytes and microglia cells.

**FIG 5:**
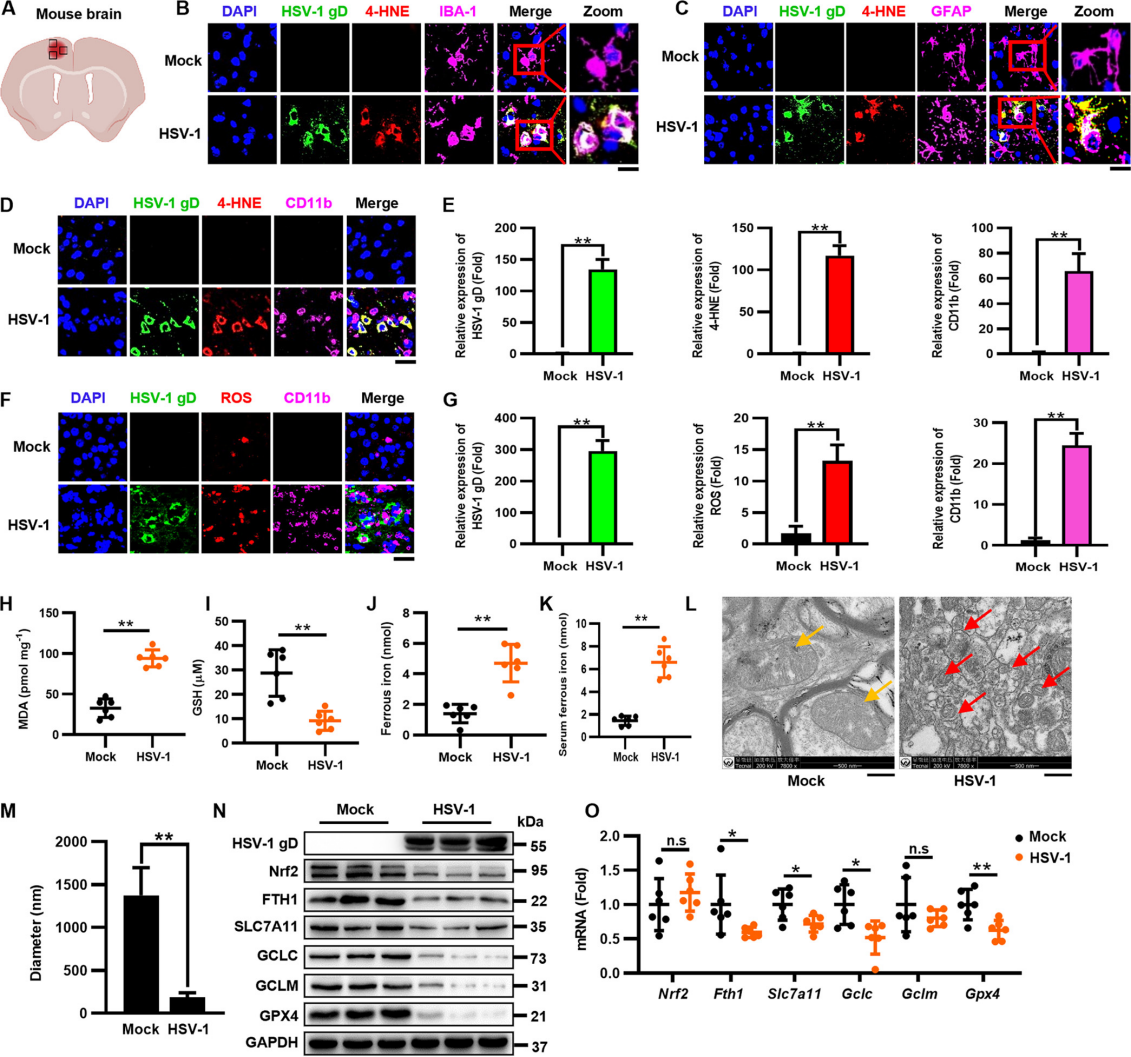
HSV-1 induces ferroptosis in murine brain astrocytes and microglia cells. Groups of 8-week-old C57BL/6 mice were i.c. injected with 1 × 10^5^ PFU HSV-1 or mock infected (*n *= 6 for each group). Mice were euthanized at 5 dpi, and brains were extracted. (A) Field of representative images of mouse brains. (B and C) The cerebral cortex sections of brains from infected or mock-infected mice were fixed and subjected to immunostaining with anti-HSV-1 gD antibody (green), the 4-HNE probe (red), anti-IBA-1 antibody (pink), and anti-GFAP antibody (pink). DAPI was used for nuclear staining (blue). Representative images were acquired using fluorescence microscopy. Bars, 20 μm. (D to G) The cerebral cortex sections of the brains from infected or mock-infected mice were fixed and subjected to immunostaining with anti-HSV-1 gD antibody (green), the 4-HNE probe (red), the ROS probe (red), and anti-CD11b antibody (pink). DAPI was used for nuclear staining (blue). Representative images were acquired using fluorescence microscopy. Bars, 20 μm. The relative expression of the indicated fluorescence signals was quantified using ImageJ software. Data represent means ± SD. **, *P < *0.01 (as measured by an unpaired *t* test). (H to J) The intracellular levels of MDA, Fe^2+^, and GSH in each group of brain lysates were measured by an MDA assay (H), an iron assay (I), and a GSSG/GSH quantification kit (J). (K) The intracellular levels of Fe^2+^ in the serum of HSV-1-infected mice were measured using an iron assay quantification kit. Data represent means ± SD. **, *P < *0.01 (as measured by an unpaired *t* test). (L) The brain lysates were subjected to transmission electron microscopy at magnifications of ×7,800. The yellow arrows indicate normal mitochondria in the brains of uninfected mice, and the red ones indicate shrunken mitochondria in the brains of HSV-1-infected mice. Representative images are shown. (M) The diameter of the mitochondria was quantified. **, *P < *0.01 (as measured by an unpaired *t* test). (N) Total proteins extracted from representative samples (*n *= 3) from different groups were subjected to Western blotting with the indicated antibodies. (O) The expression levels of the indicated genes in the different groups were determined by qRT-PCR, and the relative mRNA level of each indicated gene in the different groups was defined as 1-fold. Data represent means ± SD from three repeated experiments. *, *P < *0.05; **, *P < *0.01; n.s, no significance (as measured by an unpaired *t* test).

HSV-1 is the most common sporadic cause of encephalitis worldwide (3). After establishing that HSV-1 induces ferroptosis, we sought to evaluate the physiological relevance of ferroptosis in HSV-1-caused neurological disease. It is known that encephalitis can be induced by the intracranial (i.c.) inoculation of HSV-1 in a mouse model (32). Thus, 8-week-old C57BL/6 mice were i.c. injected with 1 × 10^5^ PFU of HSV-1, and brain tissues were extracted at 5 days postinfection (dpi). We first examined whether HSV-1 infected mouse astrocytes and microglia cells and induced ferroptosis via immunofluorescence (IF) staining with anti-HSV-1 gD, anti-ionized calcium binding adaptor molecule 1 (IBA-1) (microglia marker), and anti-glial fibrillary acidic protein (GFAP) (astrocyte marker) antibodies. Moreover, 4-hydroxy-nonenal (4-HNE) (a major product of lipid peroxidation) staining was used to detect lipid peroxidation. As shown in Fig. 5A to C, HSV-1 gD (green) colocalized with IBA-1 (pink), GFAP (pink), as well as 4-HNE (red), showing that HSV-1 infection induces ferroptosis in murine brain astrocytes and microglia cells.

Because viral encephalitis is characterized by the infiltration of monocytes and macrophages into the CNS tissues (33), we further examined the colocalization of HSV-1 and monocytes/macrophages in the infected mouse brain via immunofluorescence staining with anti-CD11b antibody. Moreover, dihydroethidium (DHE) fluorescent probe staining was used to detect ROS production. As shown in Fig. 5D and E, HSV-1 gD (green) occurred along with the presence of CD11b^+^ monocytes/macrophages (pink) as well as 4-HNE signals (red), which was also coincident with the enhanced ROS production (red) (Fig. 5F and G). Moreover, lipid peroxidation in HSV-1-infected brain tissues was further confirmed by examining the intracellular MDA level (Fig. 5H). Furthermore, the intracellular Fe^2+^ level was significantly increased, while the GSH level was significantly reduced in the brain tissues of HSV-1-infected mice compared with those in uninfected mice (Fig. 5I and J). We also observed iron overload in the serum of HSV-1-infected mice (Fig. 5K). Transmission electron microscopy analysis also showed that the mitochondria in HSV-1-infected brains displayed the typical morphological features of ferroptosis (Fig. 5L and M). Besides, we found that HSV-1 infection reduced the protein level of Nrf2 but not its mRNA level in the brains of HSV-1-challenged mice (Fig. 5N and O), consistent with the data observed in cells where HSV-1 controls Nrf2 at the protein level. Correspondingly, HSV-1-enhanced Nrf2 degradation resulted in reductions in the mRNA and protein levels of Nrf2’s downstream antiferroptotic genes in the infected brains (Fig. 5N and O). The mRNA level of GCLM was also reduced, although this difference did not reach significance, probably due to individual differences.

Together, our findings uncover that HSV-1 induces ferroptosis in murine brain astrocytes and microglia cells. Moreover, the presence of ferroptosis hallmarks coincident with viral encephalitis suggests that ferroptosis contributes to HSV-1-caused encephalitis.

### HSV-1 ferroptosis-activated upregulation of PTGS2 and PGE_2_ contributes to viral encephalitis.

**FIG 6:**
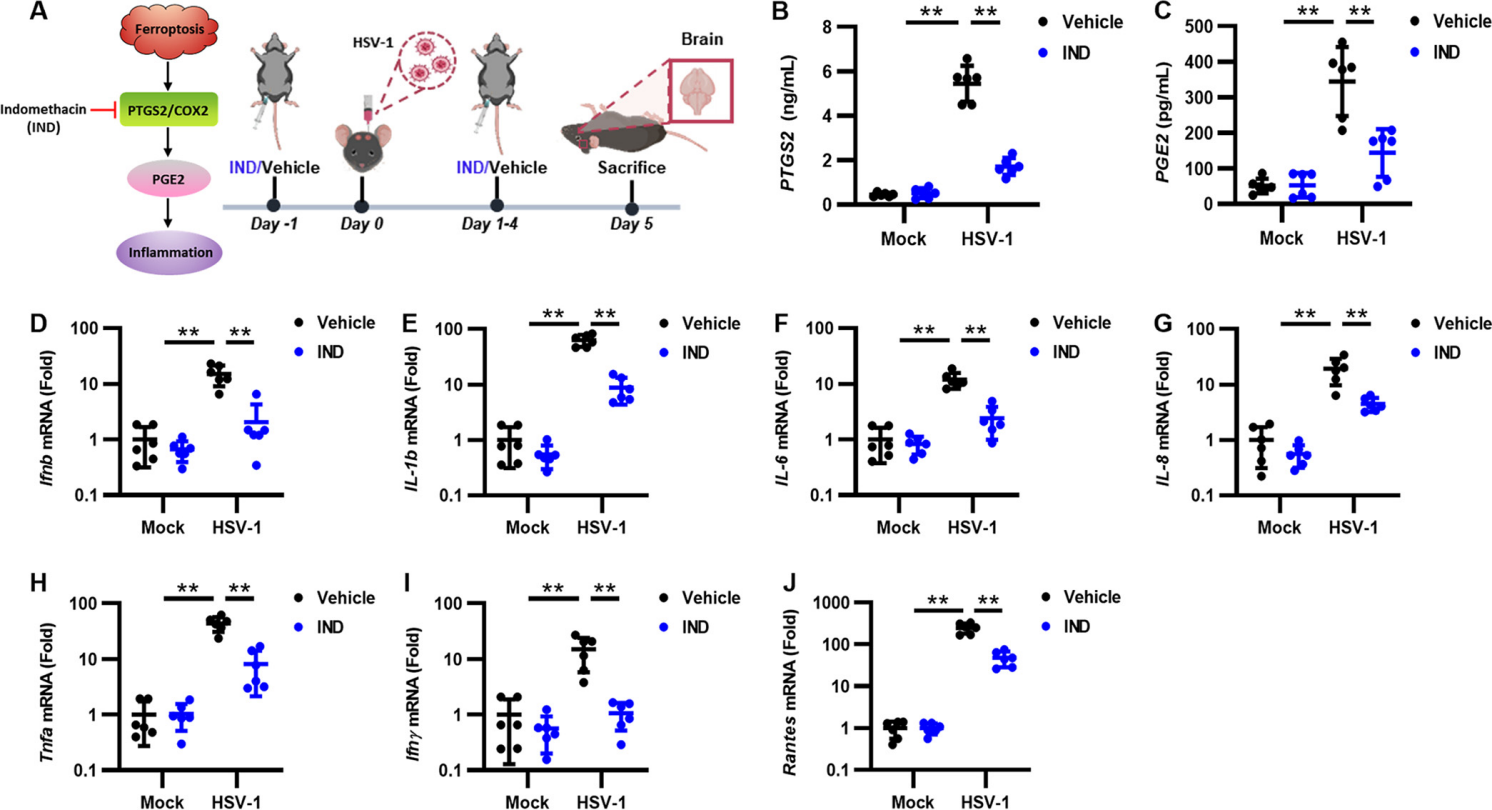
HSV-1 ferroptosis-activated upregulation of PTGS2 and PGE_2_ contributes to viral encephalitis. (A) Groups of 8-week-old C57BL/6 mice (*n *= 6 for each group) were i.p. injected with IND (10 mg/kg) or the vehicle (DMSO) 24 h before challenge with 1 × 10^5^ PFU of HSV-1 or mock infected, followed by treatment once a day for 5 days. Mice were euthanized at 5 dpi, and brains were extracted. (B and C) The levels of PTGS2 and PGE_2_ in serum were determined by ELISAs. (D to J) Total RNAs were extracted from the brains of mice from different groups and subjected to qRT-PCR to determine the expression levels of the indicated proinflammatory genes, and the relative mRNA level of each indicated gene in uninfected mice treated with the vehicle was defined as 1-fold. Data represent means ± SD. **, *P < *0.01 (as measured by two-way ANOVA).

Ferroptosis is a type of inflammatory cell death with the release of different damage-associated molecular patterns (DAMPs) and/or lipid peroxidation products (34). In addition, the activation of ferroptosis can upregulate the expression of prostaglandin-endoperoxide synthase 2 (PTGS2) (also called COX2), which metabolizes arachidonic acid (AA) to various bioactive prostaglandins (PGs) such as PGE_2_, an important mediator promoting inflammation (35). Therefore, ferroptosis can exert proinflammatory effects by accelerating the metabolism of AA and promoting the release of PGE_2_. After determining that HSV-1 induces ferroptosis in the murine brain, we examined whether the ferroptosis-induced activation of the PTGS2-PGE_2_ axis plays a role in HSV-1 encephalitis. Thus, C57BL/6 mice were intraperitoneally (i.p.) injected with indomethacin (IND), a PTGS1/PTGS2 inhibitor, at a dose of 10 mg/kg of body weight or with the vehicle (DMSO) 24 h before challenge with HSV-1 (1 × 10^5^ PFU), followed by treatment once a day, and serum and brain tissues were then extracted at 5 dpi (Fig. 6A). An enzyme-linked immunosorbent assay (ELISA) of the brain homogenates confirmed that HSV-1 infection indeed resulted in the significant upregulation of PTGS2 and PGE_2_ (Fig. 6B and C). IND, but not vehicle, treatment substantially reduced the expression levels of PTGS2 and PGE_2_ in HSV-1-infected mice (Fig. 6B and C). Consistently, the expression levels of proinflammatory genes such as interferon beta (IFN-β), interleukin-1β (IL-1β), IL-6, IL-8, TNF-α, IFN-γ, and RANTES were significantly reduced when infected mice were treated with IND (Fig. 6D to J). These findings indicate that the HSV-1 ferroptosis-activated upregulation of PTGS2 and PGE_2_ plays an important role in virus-induced inflammation and encephalitis.

### Inhibition of ferroptosis protects mice against HSV-1 encephalitis.

We sought to determine if inhibiting ferroptosis would rescue HSV-1-caused encephalitis in mice. Thus, C57BL/6 mice were i.p. injected with Fer-1 at doses of 5, 10, and 20 mg/kg or with the vehicle (DMSO) 24 h before challenge with HSV-1 (1 × 10^5^ PFU), followed by treatment once a day. Our results showed that treatment with Fer-1 increased the survival of HSV-1-infected mice in a dose-dependent manner (Fig. S9A). Because 10 mg/kg Fer-1 could rescue approximately one-half of the HSV-1-infected mice, we used this dosage for the subsequent experiments.

10.1128/mbio.02370-22.9 FIG S9 Fer-1 treatment enhances the protein level of Nrf2 and the mRNA levels of Nrf2’s downstream genes in the brains of infected mice. (A) Groups of 8-week-old C57BL/6 mice (*n *= 9 for each group) were i.p. injected with Fer-1 (5, 10, or 20 mg/kg) or the vehicle (DMSO) 24 h before challenge with 1 × 10^5^ PFU of HSV-1 or mock infected, followed by treatment once a day. Survival was recorded daily. (B to D) The intracellular levels of Fe^2+^, MDA, and GSH in each group of brain lysates were measured using an iron assay (B), an MDA assay (C), and a GSSG/GSH quantification kit (D). Data represent means ± SD. **, *P < *0.01 (as measured by two-way ANOVA). (E and F) The cerebral cortex sections of the brains of mice from different groups were fixed and subjected to immunostaining with anti-HSV-1 gD antibody (green), the ROS probe (red), and anti-CD11b antibody (pink). DAPI was used for nuclear staining (blue). Representative images were acquired using fluorescence microscopy. Bar, 20 μm. The relative expression of the indicated fluorescent signals was quantified using ImageJ software. **, *P < *0.01 (as measured by two-way ANOVA). (G) Total proteins extracted from representative samples (*n *= 2) from different groups were subjected to Western blotting with the indicated antibodies. (H to L) Total RNAs were extracted from the brains of mice from different groups and subjected to qRT-PCR, and the relative mRNA level of each indicated gene in uninfected mice treated with the vehicle was defined as 1-fold. Data represent means ± SD. *, *P < *0.05; **, *P < *0.01; n.s., no significance (as measured by two-way ANOVA). (M and N) The cerebral cortex sections of mouse brains from different groups were fixed and subjected to IHC staining with neurofilament antibody (brown). Representative images were acquired using light microscopy. Bar, 20 μm. The area of the neurofilament fraction was quantified using ImageJ software, and the relative level of neurofilament signals in the brains of uninfected mice treated with the vehicle was defined as 1-fold. **, *P < *0.01 (as measured by two-way ANOVA). Download FIG S9, TIF file, 0.2 MB. Copyright © 2022 Xu et al. 2022 Xu et al. https://creativecommons.org/licenses/by/4.0/ This content is distributed under the terms of the Creative Commons Attribution 4.0 International license.

**FIG 7:**
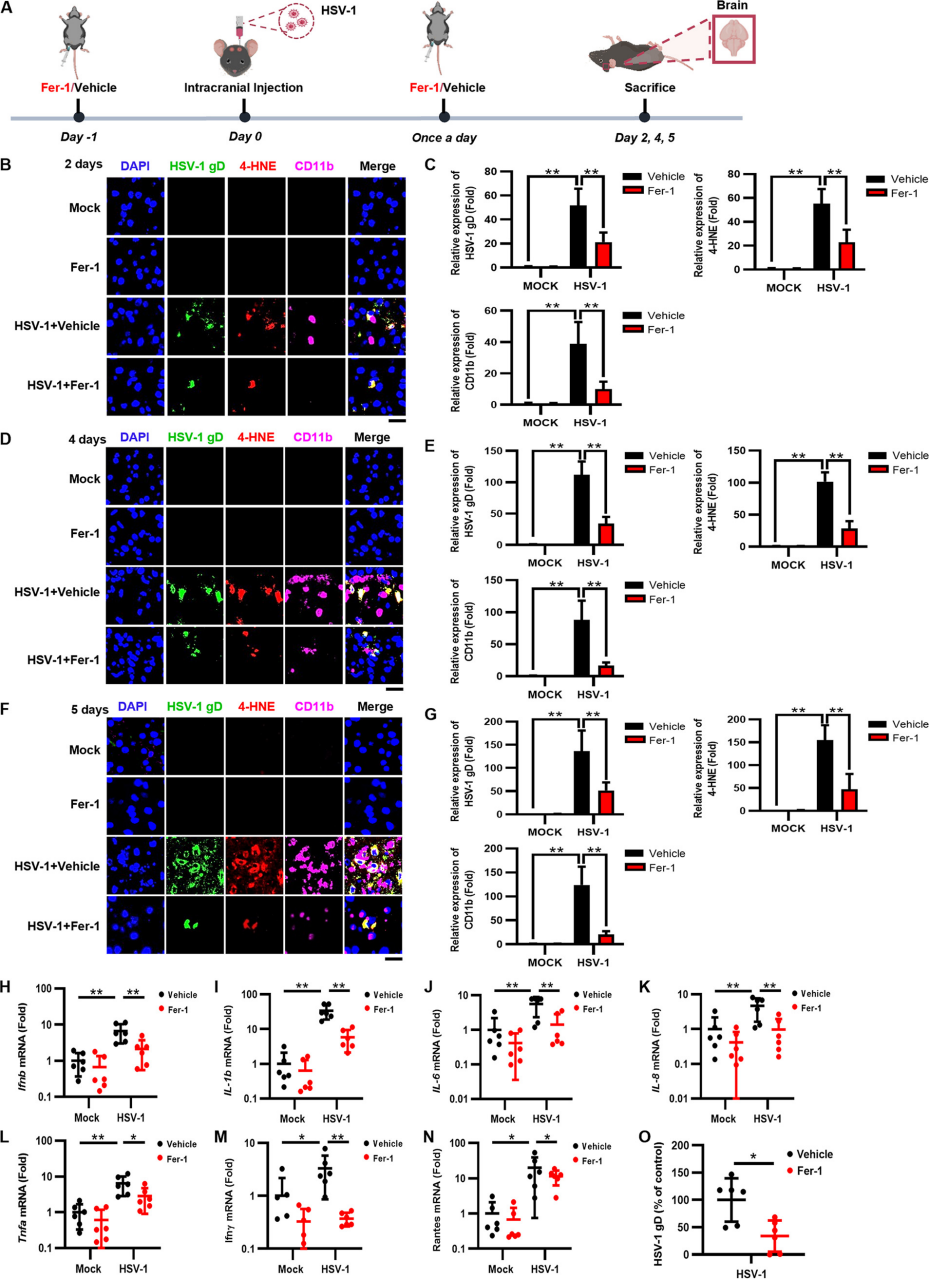
Fer-1 treatment protects mice against HSV-1 encephalitis. (A) Groups of 8-week-old C57BL/6 mice (*n *= 6 for each group) were i.p. injected with Fer-1 (10 mg/kg) or the vehicle (DMSO) 24 h before challenge with 1 × 10^5^ PFU of HSV-1 or mock infected, followed by treatment once a day. Mice were euthanized at 2, 4, or 5 dpi, and brains were extracted. (B to G) The cerebral cortex sections of the brains at different time points were fixed and subjected to immunostaining with anti-HSV-1 gD antibody (green), the 4-HNE probe (red), and anti-CD11b antibody (pink). DAPI was used for nuclear staining (blue). Representative images were acquired using fluorescence microscopy. Bars, 20 μm. The relative expression levels of the indicated fluorescence signals were quantified using ImageJ software. **, *P < *0.01 (as measured by two-way ANOVA). (H to O) Total RNAs were extracted from the brains of mice from different groups and subjected to qRT-PCR to determine the expression levels of the indicated proinflammatory genes and HSV-1 gD, and the relative mRNA level of each indicated gene in uninfected mice treated with the vehicle was defined as 1-fold, or the relative RNA level of HSV-1 gD in infected cells treated with the vehicle was defined as 100%. Data represent means ± SD. *, *P < *0.05; **, *P < *0.01 (as measured by two-way ANOVA).

We further examined the mechanisms behind the therapeutic effect of Fer-1 by extracting brain tissues from HSV-1-infected mice treated with 10 mg/kg Fer-1 at 2, 4, and 5 dpi (Fig. 7A). Immunofluorescence staining showed that the staining signals of HSV-1 gD, 4-HNE, and CD11b^+^ monocytes/macrophages were progressively increased with increasing infection times (Fig. 7B to G), confirming that HSV-1-induced ferroptosis is tightly associated with the process of viral encephalitis. In contrast, Fer-1 treatment inhibited the increases in lipid peroxidation and monocyte infiltration as well as the spread of HSV-1 (Fig. 7B to G).

Moreover, Fer-1 treatment substantially reduced the Fe^2+^/MDA levels and increased the GSH levels in HSV-1-infected brains (Fig. S9B to D). Consistently, the ROS signal was also significantly reduced in the Fer-1 therapeutic group compared with that in the vehicle-treated group (Fig. S9E and F). In addition, Fer-1 treatment also restored the expression levels of antiferroptotic genes in HSV-1-infected brains (Fig. S9G to L). Moreover, the expression levels of proinflammatory genes were significantly reduced when infected mice were treated with Fer-1 (Fig. 7H to N), showing that both ferroptosis and inflammation were inhibited by Fer-1 in the infected brain tissues. Fer-1 treatment inhibited viral replication in HSV-1-infected brains (Fig. 7O), consistent with the data obtained using cell lines. Moreover, we examined the therapeutic effect of Fer-1 on HSV-1-caused tissue damage via immunohistochemistry (IHC) staining with antineurofilament antibody. Upon HSV-1 infection, the integrity of neurofilaments was disrupted, while Fer-1 treatment maintained the integrity of neurofilaments in the cerebral cortex of infected mice (Fig. S9M and N), in agreement with the results showing that ferroptosis, monocyte infiltration, oxidative damage, and the expression levels of proinflammatory genes were inhibited in the Fer-1 therapeutic group. Together, these findings indicate that the inhibition of ferroptosis by Fer-1 effectively alleviates HSV-1-caused encephalitis in mice.

**FIG 8:**
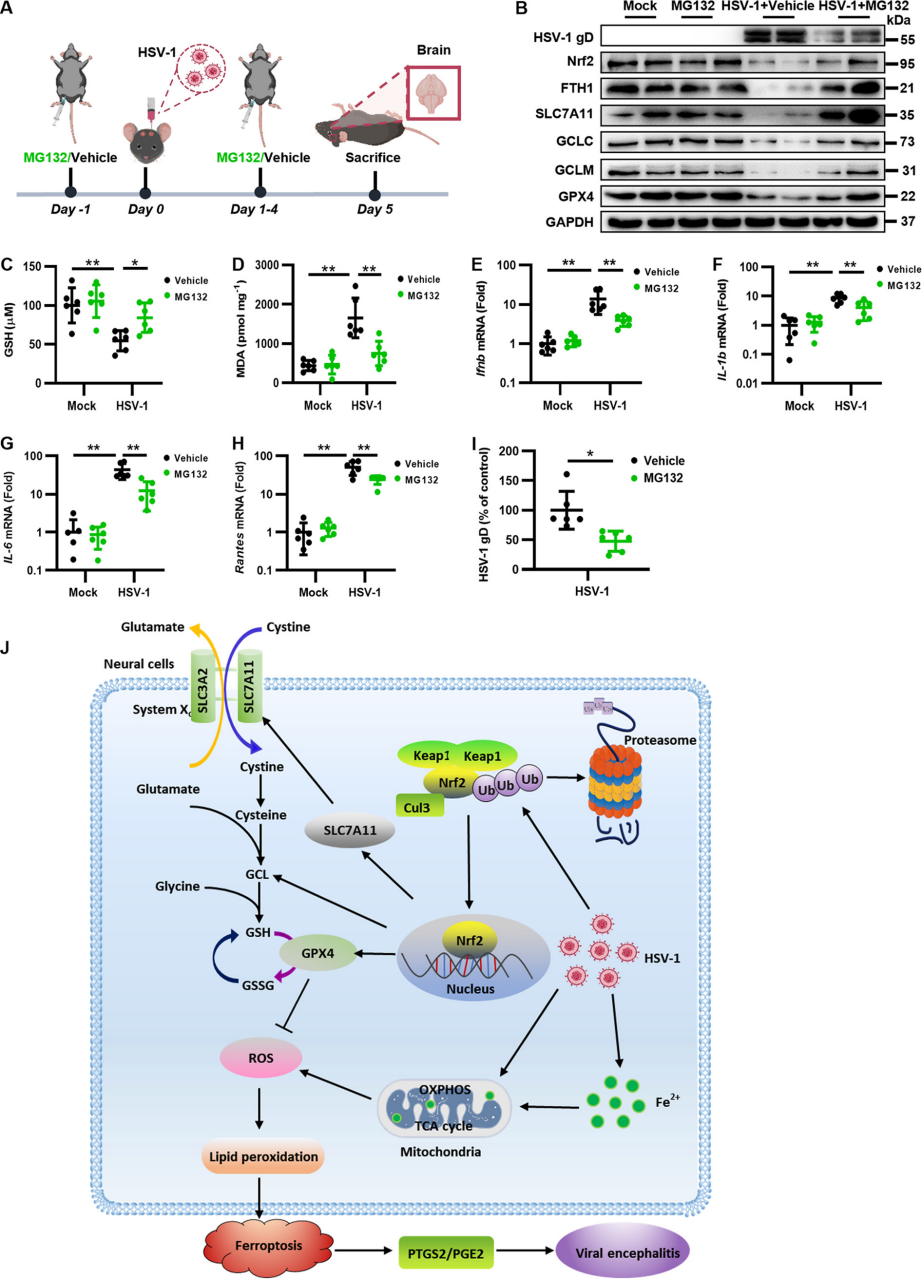
MG132 treatment protects mice against HSV-1 encephalitis. (A) Groups of 8-week-old C57BL/6 mice (*n *= 6 for each group) were i.p. injected with MG132 (10 mg/kg) or the vehicle (DMSO) 24 h before challenge with 1 × 10^5^ PFU of HSV-1 or mock infected, followed by treatment once a day for 5 days. Mice were euthanized at 5 dpi, and brains were extracted. (B) Total proteins extracted from representative samples (*n *= 2) from different groups were subjected to Western blotting with the indicated antibodies. (C and D) The intracellular levels of GSH and MDA in each group of brain lysates were measured using a GSSG/GSH quantification kit (C) and an MDA assay (D). Data represent means ± SD. *, *P < *0.05; **, *P < *0.01 (as measured by two-way ANOVA). (E to I) Total RNAs were extracted from the brains of mice from different groups and subjected to qRT-PCR to determine the expression levels of the indicated proinflammatory genes and HSV-1 gD, and the relative mRNA level of each indicated gene in uninfected mice treated with the vehicle was defined as 1-fold, or the relative RNA level of HSV-1 gD in infected cells treated with the vehicle was defined as 100%. Data represent means ± SD. *, *P < *0.05; **, *P < *0.01 (as measured by two-way ANOVA). (J) Model of the possible mechanism underlying the induction of ferroptosis by HSV-1 in neural cells.

To further confirm the therapeutic effect of inhibiting ferroptosis on HSV-1 encephalitis, we focused on Nrf2 signaling as HSV-1 can promote ferroptosis by targeting Nrf2. Because HSV-1-enhanced Nrf2 degradation can be effectively attenuated by MG132 in the infected cells, we then examined the physiological role of MG132 in mice challenged with HSV-1. Thus, C57BL/6 mice were i.p. injected with MG132 at a dose of 10 mg/kg or with the vehicle 24 h before challenge with HSV-1 (1 × 10^5^ PFU), followed by treatment once a day for 5 days, and the brain tissues were extracted at 5 dpi (Fig. 8A). Our results showed that MG132 treatment reversed the downregulated protein level of Nrf2 as well as the levels of its downstream antiferroptotic genes in the brain tissues of HSV-1-infected mice (Fig. 8B). Correspondingly, the level of GSH was significantly increased and the level of lipid peroxidation was significantly reduced in the MG132 therapeutic group compared with those in the vehicle-treated group (Fig. 8C and D). These results were consistent with the findings that MG132 treatment inhibited HSV-1-enhanced Nrf2 degradation and ferroptosis in neural cells (Fig. 4). Moreover, the inhibition of lipid peroxidation and ferroptosis by MG132 was coincident with substantial reductions in the expression levels of proinflammatory genes in HSV-1-infected mice (Fig. 8E to H). In addition, MG132 treatment also inhibited viral replication in HSV-1-infected brains (Fig. 8I). These results indicate that the inhibition of HSV-1-enhanced Nrf2 degradation by MG132 protected mice against viral encephalitis.

In summary, our findings indicate that the inhibition of ferroptosis has effective *in vivo* therapeutic effects on HSV-1-caused encephalitis.

## DISCUSSION

Ferroptosis is frequently found to be induced under pathological conditions, including neurotoxicity, neurodegenerative diseases, acute renal failure, hepatic and heart ischemia/reperfusion injury, and cancer cell death (36), while the role of ferroptosis in viral infection and pathogenesis is poorly understood. Here, we demonstrated that HSV-1 can induce ferroptosis in cells and *in vivo*. Keap1-dependent Nrf2 degradation enhanced by HSV-1 contributed to the promotion of ferroptosis. Moreover, HSV-1-induced ferroptosis played an important role in the development of viral encephalitis in mice, and the upregulation of PTGS2 and PGE_2_ activated by ferroptosis contributes to encephalitis (Fig. 8J). Importantly, the inhibition of ferroptosis substantially alleviated neuropathological damage and inflammation in the brains of HSV-1-infected mice. Our findings provide compelling evidence that HSV-1-induced ferroptosis plays an important role in viral neuropathogenesis, and the inhibition of ferroptosis is a promising immunotherapeutic strategy to treat HSV-1 infection and encephalitis.

We showed that HSV-1 infection resulted in metabolic reprogramming from OXPHOS toward aerobic glycolysis (Warburg effect) and that this contributed to the production of ROS and lipid peroxidation. Our findings are consistent with previous results showing that HSV-1 activates glycolysis by regulating 6-phosphofructo-1-kinase (37) and that HSV-1 induces oxidative stress and the release of lipid peroxidation by-products in mouse neural cells (38). Concurrently, the antioxidative response, i.e., the biosynthesis of GSH, was interrupted by HSV-1, as evidenced by the findings that both the GSH level and the GSH/GSSG ratio were decreased along with increases in ROS production, lipid peroxidation, and cell death in HSV-1-infected cells. Moreover, the inhibition of ferroptosis by Fer-1 restored the inhibitory effect of HSV-1 infection on the GSH level and the GSH/GSSG ratio. These results indicate that the antioxidative response, particularly the biosynthesis of GSH, plays a critical role in counteracting HSV-1-induced ferroptosis. The observation that supplementation with exogenous GSH can efficiently reduce HSV-1-induced ROS and ferroptosis also supports this notion. Based on these findings, we speculate that ROS production triggered by an HSV-1-directed metabolic switch and the interruption of GSH biosynthesis act together to promote ferroptosis in HSV-1-infected neural cells.

The antioxidant transcription factor Nrf2 is a key negative player in ferroptosis (8). Almost all genes implicated in antiferroptosis thus far have been considered to be transcriptionally regulated by Nrf2, including iron metabolism, glutathione biosynthesis, and NADPH regeneration, which is critical for GPX4 activity (39). Under normal conditions, Nrf2 is located in the cytosol and interacts with its repressor Keap1, which directs the ubiquitination and proteasomal degradation of Nrf2 (40). Under oxidative stress, Nrf2 is released from the Keap1 binding site and is rapidly transferred to the nucleus, activating the transcription of antioxidant-related genes to balance oxidative stress. We showed that HSV-1 enhanced the Keap1-dependent ubiquitination and degradation of Nrf2, which resulted in substantial reductions in the expression levels of antioxidative genes, thereby disturbing cellular redox homeostasis and promoting ferroptosis. In addition, upregulating Nrf2 via inhibiting its proteasomal degradation or overexpressing Nrf2 itself effectively alleviated the process of HSV-1-induced ferroptosis in cells and neuropathogenic damage in HSV-1-infected mice. In addition, Fer-1 treatment reversed the HSV-1-induced reduction in the expression of antioxidative genes. We speculate that the inhibition of HSV-1-induced ROS accumulation/oxidative stress by Fer-1 may prevent cytoplasmically localized Nrf2 from being released from Keap1 and being transferred to the nucleus to transcribe antioxidative genes. Together, our findings uncover that Nrf2 participates in counteracting HSV-1 infection and viral pathogenesis. Consistently, single-cell RNA sequencing analysis has shown that Nrf2 activity is associated with a lower efficiency of HSV-1 infection, and the treatment of infected cells with Nrf2 agonists can restrict HSV-1 replication (41). Moreover, a previous report has shown that the overexpression of Nrf2 in a mouse model promotes neuronal survival in neurodegeneration and acute nerve damage (42).

Previous studies have shown that proteasome functions are necessary for multiple viral events during the HSV life cycle (43–48), and MG132 and other proteasome inhibitors have been reported to inhibit these proteasome-dependent events (49, 50). Here, we showed that MG132 treatment effectively reversed Keap1-dependent Nrf2 degradation and the reduced expression of downstream antioxidant genes in HSV-1-infected cells and mice, indicating that the inhibition of Nrf2 degradation by MG132 contributes to the alleviation of HSV-1-induced ferroptosis and pathogenesis. Thus, our findings uncovered that MG132 can confer an antiferroptosis effect via its proteasome-inhibiting activity in HSV-1-infected cells.

HSV-1 infection of the brain causes devastating necrotizing encephalitis. The intracranial delivery of HSV-1 in a mouse model has been found to induce the robust activation of brain microglial cells and the production of proinflammatory cytokines, which leads to exacerbated inflammation and tissue damage or even death (51). We showed that HSV-1 encephalitis occurred coincident with the hallmarks of ferroptosis and oxidative damage in murine brain astrocytes and microglia cells. Moreover, the observations that ferroptosis and monocyte infiltration were progressively increased with increasing durations of infection confirm that HSV-1-induced ferroptosis is tightly associated with the process of viral encephalitis. More importantly, the inhibition of ferroptosis by either Fer-1 treatment or the suppression of HSV-1-enhanced Nrf2 degradation effectively alleviated HSV-1-caused encephalitis in a mouse model. HSV-1 can also cause other types of cell death in CNS cells (i.e., apoptosis and necroptosis). We showed that Fer-1 treatment had little effect on apoptosis and necroptosis in HSV-1-infected cells, indicating that the rescuing effect of Fer-1 on HSV-1 infection was indeed via inhibiting ferroptosis. Together, these findings demonstrate that HSV-1-induced ferroptosis plays an important role in the development of viral encephalitis. Our findings are in accordance with previous observations that ferroptosis is involved in the development of different inflammatory diseases, including atherosclerosis, stroke, intracerebral hemorrhage, and ischemia/reperfusion injury (36). Indeed, ferroptosis is considered a type of inflammatory cell death, which is caused by lipid peroxidation-directed cell membrane damage (4). Ferroptotic cell death results in the release of different DAMPs (e.g., high-mobility-group box 1) or lipid peroxidation products (e.g., 4-HNE) (52), which can further activate inflammation and the corresponding oxidative injury. Moreover, ferroptosis can exert a proinflammatory effect by accelerating the metabolism of AA and promoting the release of PGE_2_ (34). Our findings indicate that the HSV-1 ferroptosis-induced upregulation of PTGS2 and PGE_2_ directly participates in virus-induced inflammation. Besides, our findings suggest that HSV-1-induced ferroptosis and ROS may enable efficient viral replication, as evidenced by the finding that both viral yields and cell death were reduced when HSV-1-infected cells were treated with Fer-1 or GSH. A possible explanation is that an increase in the intracellular ROS level can result in lipid peroxidation and cell death, which is beneficial for virion release and systemic viral spread. Moreover, the increased ROS levels are tightly associated with virus-induced metabolic reprogramming, which has been found to support the replication of various viruses (53). Collectively, considering that ferroptosis facilitates HSV-1 replication, the exacerbation of inflammation, and tissue damage, we conclude that ferroptosis is a pivotal factor contributing to the pathogenesis of HSV-1 infection.

A growing list of viruses have been reported to induce ferroptosis. For example, Newcastle disease virus induces ferroptotic cell death in tumor cells through nutrient deprivation and ferritinophagy (54). Hepatitis A virus 3C induces ferroptosis in human cells depending on its protease activity (55). Epstein-Barr virus dynamically sensitizes B cells to ferroptosis for latency (56). In addition, a number of human enteroviruses and coronaviruses induce ferroptosis via acyl-coenzyme A (CoA) synthetase long-chain family member 4 (ACSL4) (57), and murine coronavirus can trigger ferroptosis through ACSL1 (58). Moreover, oxidative stress and lipid peroxidation leading to exacerbated inflammation and tissue damage have been considered important in the pathogenesis of different viruses (59). Based on these recent reports and our findings in this study, we postulate that ferroptosis is a common feature of infection by diverse viruses and that it is tightly correlated with the pathogenesis of different viral diseases. In support of this, recent evidence has shown that severe acute respiratory syndrome coronavirus 2 (SARS-CoV-2) triggers ferroptosis in cells of multiple organs, thereby contributing to multiorgan damage (60, 61). Thus, ferroptosis can be considered an emerging target in viral diseases. In this study, we showed that Fer-1 treatment effectively alleviates HSV-1-caused encephalitis in a mouse model. Moreover, the application of *N*-acetylcysteine, an ROS inhibitor, to alleviate ferroptosis improves the outcomes of severe coronavirus disease 2019 (COVID-19) patients in clinical trials (62). Besides, NAD^+^ and its intermediate, both of which can reduce the intracellular levels of ROS (63), have recently been found to alleviate pathological damage in the lungs of SARS-CoV-2-infected mice (64). Ferroptosis inhibitors have the promising potential to be further developed to treat different types of viral infections.

In conclusion, our findings uncover the interaction between HSV-1 infection and ferroptosis, shed novel light on the physiological impacts of ferroptosis on the pathogenesis of HSV-1 infection and encephalitis, and provide a promising therapeutic strategy to treat this important infectious disease with a worldwide distribution.

## MATERIALS AND METHODS

### Cells and viruses.

U373 cells (human astrocytoma cell line) were obtained from the European Collection of Authenticated Cell Cultures (ECACC) (ECACC 08061901), and HMC3 cells (human microglial cell line) were obtained from the American Type Culture Collection (ATCC) (ATCC CRL-3304). U373 and Vero cells were cultured in Dulbecco’s modified Eagle’s culture medium (DMEM; Gibco, Grand Island, NY, USA) supplemented with 10% fetal bovine serum (FBS; Gibco) at 37°C in a humidified atmosphere with 5% CO_2_. HMC3 cells were cultured in minimum essential medium (MEM; Gibco, Grand Island, NY, USA) supplemented with 10% FBS at 37°C in a humidified atmosphere with 5% CO_2_. HSV-1 strain 17 syn^+^ (a wild type strain) (GenBank accession number NC_001806) was propagated at a low MOI in Vero cells.

### Plasmids, siRNAs, and reagents.

Plasmids encoding FLAG-tagged Keap1, HA-tagged Ub, and His-tagged Nrf2 were purchased from GeneChem (Shanghai, China). Plasmids were transfected into cells using Lipofectamine 2000 reagent (Life Technologies). The small interfering RNAs (siRNAs) (nontargeting control siRNA and siRNA targeting Keap1) used in this study were synthesized by RiboBio (Guangzhou, China). The siRNAs were transfected into cells using riboFECT CP (RiboBio) according to the manufacturer’s instructions. Ferrostatin-1 (Fer-1) (catalog number S7243), RSL3 (catalog number S8155), MG132 (catalog number S2619), chloroquine (NSC-187208) (catalog number S6999), and indomethacin (IND) (NSC-77541) (catalog numberS1723) were purchased from Selleck Chemicals. Reduced l-glutathione (GSH) (catalog number HY-D0187) and staurosporine (STS) (catalog number HY-15141) were purchased from MedChemExpress (MCE). Deferoxamine (DFO), *N*-acetylcysteine (NAC), and TNF-α were purchased from Sigma-Aldrich.

### Electron microscopy.

Cell samples were fixed in 2.5% glutaraldehyde in 0.1 M sodium cacodylate buffer (pH 7.4) for 1 h and then fixed in 1% osmium tetroxide for 1 h, followed by staining with 2% uranyl acetate in maleate buffer (pH 5.2) for another 1 h. The samples were rinsed, dehydrated in an ethanol series, embedded in resin (Embed812; Electron Microscopy Sciences [EMS]), and baked overnight at 60°C. Hardened blocks were cut using a Leica Ultra Cut Ultrasonic computed tomography (UCT) instrument. Sixty-nanometer sections were collected on carbon-coated grids and contrast stained using 2% uranyl acetate and lead citrate. The grids were observed under an electron microscope (Tecnai G^2^ 20 Twin; FEI, USA).

### Cell viability and LDH release assays.

The cell counting kit 8 (CCK8) (Sigma, St. Louis, MO, USA) reagent was used to examine cell viability according to the manufacturer’s instructions. In brief, cultured cells were seeded at a density of approximately 5,000 cells/well in 96-well plates. At the end of different treatments, 10 μL per well of the CCK8 reagent was added to each well, and the mixture was incubated for 2 h. The absorbance at 450 nm was measured using a plate reader (Bio-Rad, Hercules, CA, USA). The extent of cellular injury was determined by LDH leakage using an LDH cytotoxicity detection kit (Dojindo Laboratory, Kumamoto, Japan) according to the manufacturer’s instructions. Briefly, cells were seeded at a density of approximately 5,000 cells/well in 96-well plates. At the end of different treatments, 100 μL of a fresh reaction mixture was added to each well, and the cells were incubated for 30 min. The absorbance at 490 nm was measured using a plate reader (Bio-Rad).

### Lipid peroxidation MDA assay and 4-HNE assay.

Malondialdehyde (MDA) and 4-hydroxy-nonenal (4-HNE) are the major products of lipid peroxidation (65). MDA concentrations were measured using an MDA assay kit (Sigma) according to the manufacturer’s instructions. In this assay, lipid peroxidation is determined by the reaction of MDA with thiobarbituric acid (TBA) to form a colorimetric product (532 nm), which is directly proportional to the MDA concentration. The absorbance at 532 nm was measured using a plate reader (Bio-Rad). The expression of 4-HNE in mouse brain tissues was determined using anti-4-HNE antibody (Abcam). Paraffin-embedded mouse brain tissue sections were incubated with anti-4-HNE antibody at a 1/25 dilution overnight at 4°C. Polyclonal goat anti-mouse IgG conjugated to Alexa Fluor 594 was used as the secondary antibody. The fluorescence intensity was analyzed using ImageJ software.

### Ferrous iron detection.

Ferrous iron (Fe^2+^) was measured using an iron assay kit (Sigma) and a FerroOrange probe (Dojindo Laboratory). For the iron assay kit, iron is released by the addition of an acidic buffer and then reacted with a chromogen, resulting in a colorimetric product (593 nm), which is directly proportional to the Fe^2+^ concentration. The absorbance at 593 nm was measured using a plate reader (Bio-Rad). The FerroOrange probe was used for fluorescence imaging of intracellular Fe^2+^ (54). Briefly, FerroOrange (1 mM) dispersed in serum-free medium was added to the cells, followed by incubation for 30 min at 37°C. The cells were then fixed with 4% paraformaldehyde for 45 min and permeabilized with 0.2% Triton X-100 for 20 min. After that, the cells were blocked with phosphate-buffered saline (PBS) containing 5% bovine serum albumin (BSA) for 1 h and then incubated with anti-HSV-1 gD antibody (1:1,000 in 5% BSA) overnight, followed by staining with FITC-labeled goat anti-mouse IgG (ABclonal) (1:1,000 in 5% BSA). Nuclei were stained with 4′,6-diamidino-2-phenylindole (DAPI; Beyotime) for 5 min at 37°C in the dark. Cells were photographed under a confocal microscope (A1R; Nikon, Japan). The fluorescence intensity was analyzed using ImageJ software.

### ROS measurement.

The level of intracellular ROS was determined by using a fluorometric intracellular ROS kit (Sigma) and a DCFH-DA probe (Dojindo Laboratory), respectively. For the fluorometric intracellular ROS kit, cells were seeded at a density of approximately 5,000 cells/well in 96-well plates. At the end of different treatments, the ROS detection reagent stock solution containing the cell-permeable oxidative fluorescent dye 2′,7′-dichlorodihydrofluorescein diacetate was added to the wells, followed by incubation for 1 h at 37°C. The ROS level was quantified by measuring the fluorescence intensity at wavelengths of 490 and 525 nm. For the DCFH-DA probe, cells were seeded into a 20-mm dish. At the end of different treatments, cells were stained with DCFH-DA for 30 min at 37°C. The cells were then fixed with 4% paraformaldehyde for 45 min and permeabilized with 0.2% Triton X-100 for 20 min. After that, the cells were blocked with PBS containing 5% BSA for 1 h and then incubated overnight with anti-HSV-1 gD antibody (1:1,000 in 5% BSA), followed by staining with Alexa Fluor 594-conjugated goat anti-mouse IgG (ABclonal) (1:1,000 in 5% BSA). Nuclei were stained with DAPI (Beyotime) for 5 min at 37°C in the dark. Cells were photographed under a confocal microscope (A1R; Nikon, Japan). The fluorescence intensity was analyzed using ImageJ software.

### Measurement of the intracellular level of GSH and the GSH/GSSG ratio.

The intracellular level of GSH was determined using a GSSG/GSH quantification kit (Dojindo Laboratory) according to the manufacturer’s instructions. Briefly, cells were lysed in 10 mM HCl and subjected to 2 freeze-thaw cycles, followed by treatment with 5% sodium sulfosalicylate. For GSSG measurement, a masking solution was first added to the supernatant samples. After that, the samples were transferred to a 96-well plate, and buffer solution was added to each well containing the supernatant samples, allowing incubation for 1 h at 37°C. The substrate solution and coenzyme/enzyme working solution were then added to each well, and the samples were incubated for another 10 min at 37°C. Thus, total GSH and GSSG levels and their standards were measured using a plate reader at a wavelength of 412 nm.

### Seahorse analysis of the extracellular acidification rate and the oxygen consumption rate.

The extracellular acidification rate (ECAR) and the oxygen consumption rate (OCR) were measured using a Seahorse XF-24 flux analyzer (Seahorse Biosciences, North Billerica, MA) (66). Briefly, U373 or HMC3 cells were seeded into a Seahorse 24-well tissue culture plate at a density of approximately 3.5 × 10^4^ cells/well. Prior to the assay, the medium was changed to unbuffered DMEM containing pyruvate and glutamine (pH 7.4), and cells were equilibrated for 30 min at 37°C. The ECAR was determined using a glycolysis stress test kit (injections of the compounds glucose, oligomycin, and 2-Deoxy-D-glucose [2-DG]). The OCR was determined using a cell mitochondrion stress test kit (injections of the compounds oligomycin, carbonyl cyanide p-trifluoromethoxyphenylhydrazone (FCCP), and rotenone/antimycin A). The compounds were injected during the assay, and the measurement periods for examining the OCR and ECAR were 2 h.

### Stable-isotope tracing and metabolite extraction.

U373 cells were incubated in cystine-free medium supplemented with 0.26 mM [^15^N_2_]cystine and then infected with HSV-1 (MOI = 0.1) for 12 h to isotopically monitor the biosynthesis of GSH and GSSG. For the extraction of polar metabolites, the medium was discarded, and the cell pellets were washed three times with PBS and quenched with liquid nitrogen. Precooled methanol-water (80%, vol/vol) was added to the cell pellets, and the cells were scraped off with a cell scraper into a 2-mL centrifuge tube with ring-*d*_5_-labeled-phenylalanine added as an internal standard. Next, the sample was further lysed by sonication and liquid nitrogen freeze-thawing. After centrifugation at 14,000 rpm at 4°C for 15 min, the supernatant was placed into a new centrifuge tube, followed by lyophilization for concentration. The residue was redissolved with acetonitrile-water (50:50, vol/vol) with 0.1% (vol/vol) formic acid, vortexed, and centrifuged at 14,000 rpm at 4°C for 15 min, and the supernatant was then injected into the liquid chromatography-tandem mass spectrometry (LC-MS/MS) system.

### LC-MS/MS analysis.

The LC-MS/MS system was equipped with an Exion LC system (AB Sciex) and a Zwitterionic polymer Hydrophilic Interaction Liquid Chromatography (ZIC-pHILIC) column (100 by 2.1 mm, 5 μm; Millipore) connected to a Qtrap 5500 mass spectrometer (AB Sciex). Under LC conditions, 2 μL of each sample was injected for analysis, and the flow rate was 0.2 mL/min. The column temperature and tray temperature were set to 40°C and 4°C, respectively. The mobile phases were composed of 15 mM ammonium acetate in 3 mL/L ammonium hydrate (>28%) for aqueous solution A and 90% acetonitrile for aqueous solution B. The gradient program was set as follows: 95% solution B held for 1 min, decreased to 45% in 14 min and held for 2 min, and increased to 95% in 0.5 min and held for 4.5 min. In negative-ion multiple-reaction monitoring (MRM) mode, the electrospray ionization (ESI) voltage was set to −4,500 V. The ion temperature was 500°C, and the gas concentration was 35 μL/min. The LC-MS/MS conditions were controlled by Analyst 1.7.1 software, and the final data were processed using Mutiquant 3.0.3 software. Natural isotope abundances were corrected using the Isotope Distribution Calculator website (https://www.sisweb.com/mstools/isotope.htm). Next, stable-isotope labeling was analyzed using GraphPad 8.3.0 after correcting the natural abundance. The cellular metabolome data were analyzed by MetaboAnalyst 5.0 (https://www.metaboanalyst.ca/). Statistical analysis was conducted using an unpaired *t* test.

### RNA isolation and quantitative real-time PCR.

Total RNAs were isolated using TRIzol reagent (TaKaRa) according to the manufacturer’s instructions. RNA (1 μg) was reverse transcribed using PrimeScript reverse transcriptase master mix (TaKaRa). Quantitative real-time PCR (qRT-PCR) was performed by using SYBR green PCR master mix (Bio-Rad Laboratories, Hercules, CA) with the ABI 7500 fast machine (Applied Biosystems). All values were normalized to the glyceraldehyde-3-phosphate dehydrogenase (GAPDH) mRNA level. The sequences of primers used for quantitative PCR (qPCR) analysis are listed in Table S1 in the supplemental material.

10.1128/mbio.02370-22.10 TABLE S1 Real-time PCR primers. Download Table S1, DOCX file, 0.01 MB. Copyright © 2022 Xu et al. 2022 Xu et al. https://creativecommons.org/licenses/by/4.0/ This content is distributed under the terms of the Creative Commons Attribution 4.0 International license.

### Plaque assays.

Vero cells in 24-well plates were infected with 10-fold serial dilutions of viruses. Cells were cultured at 37°C for 2 h to allow the adsorption of viruses, and the supernatant was replaced with DMEM containing 2% FBS and 1% penicillin-streptomycin with isopycnic 1% low-melting-point agarose (Sigma). After incubation at 37°C for 48 h, cells were fixed with cold absolute methanol at 4°C for 1 h and then stained with 1% crystal violet.

### Western blotting and antibodies.

Cell samples and fresh brain tissues were all lysed by using radioimmunoprecipitation assay (RIPA) buffer (50 mM Tris [pH 8.0], 150 mM sodium chloride, 1% Triton X-100, 0.5% sodium deoxycholate, 0.1% sodium dodecyl sulfate) with 1% protease and phosphatase inhibitor cocktail (Roche). The concentration of total proteins was examined using a Pierce bicinchoninic acid (BCA) assay kit (Invitrogen). Equal amounts of protein lysates (20 μg) were separated on SDS-PAGE gels and transferred to polyvinylidene fluoride membranes (Millipore, Germany). The blots were incubated with primary antibodies in 5% nonfat milk in Tris-buffered saline (TBS) with 0.05% Tween 20 (TBST) overnight at 4°C. The membranes were washed in TBST and incubated with horseradish peroxidase (HRP)-conjugated secondary antibodies at room temperature for 1 h. Proteins were detected by chemiluminescence using ECL (Bio-Rad) in a Bio-Rad ChemiDoc imager and quantified using ImageJ software. The antibodies used for Western blotting are as follows: anti-HSV-1 gD, anti-Nrf2 (phospho-S40), and anti-GPX4 were obtained from Abcam; anti-Nrf2 and anti-Keap1 were obtained from Proteintech; anti-GCLC was obtained from Santa Cruz Biotechnology; anti-GCLM was obtained from GeneTex; and anti-FTH1, anti-xCT/SLC7A11, anti-ubiquitin (clone P4D1), anti-ubiquitin-K48, anti-ubiquitin-K63, anti-His tag (clone 27E8), anti-MLKL, anti-p-MLKL, anti-RIPK3, anti-p-RIPK3, and anti-GAPDH were obtained from Cell Signaling Technology (CST).

### Coimmunoprecipitation.

For the coimmunoprecipitation (co-IP) assay, cells were harvested and then lysed with IP buffer (20 mM Tris-HCl [pH 7.4], 150 mM NaCl, 10% glycerol, 2 mM EDTA, 0.5% Nonidet P-40, 0.5% Triton X-100) with 1% protease and phosphatase inhibitor (Roche). The insoluble component was removed by centrifugation at 12,000 × *g* for 10 min at 4°C, and the supernatant was collected. For each sample, 600 μL of the protein lysate was incubated with 1 μg of antibody and 30 μL of protein A/G magnetic beads (MCE) overnight at 4°C. The beads were washed 3 times with 1 mL of IP buffer, and the precipitates were then detected by Western blotting.

### Apoptosis detection assay.

Cell apoptosis was determined by using an annexin V-FITC apoptosis detection kit (catalog number C1062M; Beyotime). Briefly, 195 μL of an annexin V-FITC binding solution was added to resuspend the cells gently. Next, 5 μL of annexin V-FITC and 10 μL of a propidium iodide (PI) staining solution were added, followed by gentle mixing and incubation at room temperature in the dark for 10 to 20 min. Flow cytometry analysis was conducted within 1 h. Early apoptosis was defined as annexin V-FITC singly positive cells (third quadrant [Q3]), and late apoptosis was defined as annexin V-FITC and PI doubly positive cells (Q2); the percentage of apoptotic cells was measured as the sum of Q2 and Q3. This gating strategy was applied to all flow cytometry analyses in this study. The flow cytometry assays were performed using an LSRFortessa X-20 cell analyzer (BD Biosciences), and the data were analyzed using FlowJo v10.6.2.

### Animal studies.

Eight-week-old C57BL/6 mice (Experimental Animal Center, Wuhan Institute of Virology) were housed under specific-pathogen-free (SPF) conditions in individually ventilated cages. Mice were intracranially (i.c.) injected with 10 μL of DMEM containing 1 × 10^5^ PFU/mL of HSV-1 with a 50-μL gastight microsyringe (Hamilton, Reno, NV, USA). Fer-1 (5, 10, or 20 mg/kg), IND (10 mg/kg), or MG132 (10 mg/kg) was administered by intraperitoneal (i.p.) injection 24 h before viral infection, followed by treatment once a day. Mice were euthanized 2, 4, and 5 days after viral infection to obtain brain tissues for subsequent analyses. All experiments using animals were reviewed and approved by the Institutional Animal Care and Use Committee at the Wuhan Institute of Virology, Chinese Academy of Sciences, and were performed in accordance with the Guide for the Care and Use of Laboratory animals published by the Wuhan Institute of Virology.

### Immunohistochemistry and immunofluorescence staining.

Briefly, tissue samples were collected, mounted onto slides from paraffin blocks (5-μm sections), and then deparaffinized in xylene, followed by hydration in a methanol gradient (100%, 95%, 70%, and 50%). Slide samples were treated and 10 mM citrate buffer (pH 6.0) containing 3% H_2_O_2_ for antigen retrieval and then incubated with 5% BSA for 30 min. The slides were incubated with the primary antibody at 4°C overnight and then incubated with the biotinylated secondary antibody for 30 min. After that, an avidin-biotin complex kit (Dako/Agilent Technologies, Santa Clara, CA, USA) was used for an additional 30 min, and 3,3′-diaminobenzidine tetrahydrochloride hydrate (DAB) containing 5% H_2_O_2_ was used as a chromogen. Hematoxylin and eosin (H&E) staining was performed and visualized by microscopy (Olympus, Tokyo, Japan). The antibodies used are as follows: mouse anti-neurofilament L (clone DA2) (CST, USA), anti-4-HNE (Abcam), anti-CD11b (Abcam), anti-IBA-1 (CST), and anti-GFAP (CST). The intensity of immunohistochemistry (IHC) or immunofluorescence (IF) staining was analyzed using ImageJ software.

### Enzyme-linked immunosorbent assay.

Serum was collected and assayed to determine the levels of PTGS2 and PGE_2_ using a mouse PTGS2/COX2 enzyme-linked immunosorbent assay (ELISA) kit and a mouse PGE_2_ ELISA kit, respectively, according to the manufacturer’s instructions (Fine Test, China).

### Quantification and statistical analysis.

Statistical analyses were conducted using GraphPad Prism 8.3.0. (GraphPad Software Inc., San Diego, CA). Data are shown as means ± standard deviations (SD), and statistical significance was evaluated using an unpaired *t* test or one-way or two-way analysis of variance (ANOVA), as indicated in the figure legends. A *P* value of <0.05 was considered to be statistically significant.
