# Supplementary material for: Herpes Simplex Virus 1-Induced Ferroptosis Contributes to Viral Encephalitis
Source: mBio. 2022 Dec 12;14(1):e02370-22. doi: 10.1128/mbio.02370-22 (PMC9973258; doi:10.1128/mbio.02370-22)
Supplement: TABLE S1 [file mbio.02370-22-s0010.docx]

**Table S1. Real-time PCR primers.**

| Genes | Primer Sequence 5’ to 3’ |
| --- | --- |
| HSV-1 gD-F | ATCCGAACGCAGCCCCGC |
| HSV-1 gD-R | TCTCCGTCCAGTCGTTTAT |
| Nrf2-F | CATCCAGTCAGAAACCAGTGG |
| Nrf2-R | GCAGTCATCAAAGTACAAAGCAT |
| FTH1-F | CTGCGCCCTTCTGGAAAATG |
| FTH1-R | GCAACCCCAGGATTTCAGGA |
| SLC7A11-F | ACAGGGATTGGCTTCGTCAT |
| SLC7A11-R | GGCAGATTGCCAAGATCTCAA |
| GCLC-F | GGAGGCTACTTCTGTACTA |
| GCLC-R | GGAGGCTACTTCTGTACTA |
| GCLM-F | GGCACAGGTAAAACCAAATAGTAAC |
| GCLM-R | CAAATTGTTTAGCAAATGCAGTCA |
| GPX4-F | CAGTGAGGCAAGACCGAAGT |
| GPX4-R | CCGAACTGGTTACACGGGAA |
| GAPDH-F | GGTGGTCTCCTCTGACTTCAACA |
| GAPDH-R | GTTGCTGTAGCCAAATTCGTTGT |
| mouse Nox-1-F | TCTCCAGCCTATCTCATCCTGA |
| mouse Nox-1-R | GCTGCATACATCACTGTCATGTT |
| mouse Cox-2-F | TGGAGCCCGTGCTGCTCTGTC |
| mouse Cox-2-R | GCCTGGCAAGTCTTTAACCTCACAGC |
| mouse Ifnb-F | ATGAGTGGTGGTTGCAGGC |
| mouse Ifnb-R | TGACCTTTCAAATGCAGTAGATTCA |
| mouse IL-1b-F | CCTCTGATGGGCAACCACTT |
| mouse IL-1b-R | TTCATCCCCCACACGTTGAC |
| mouse IL-6-F | ACAGAAGGAGTGGCTAAGGA |
| mouse IL-6-R | CGCACTAGGTTTGCCGAGTA |
| mouse IL-8-F | CCTCTGATGGGCAACCACTT |
| mouse IL-8-R | TTCATCCCCCACACGTTGAC |
| mouse Tnfa-F | TCACTGGAGCCTCGAATGTC |
| mouse Tnfa-R | GTGAGGAAGGCTGTGCATTG |
| mouse Ifnγ-F | GACTGTGATTGCGGGGTTGT |
| mouse Ifnγ-R | GGCCCGGAGTGTAGACATCT |
| mouse Rantes-F | GCAGCAAGTGCTCCAATCTT |
| mouse Rantes-R | CAGGGAAGCGTATACAGGGT |
| mouse Nrf2-F | TAGATGACCATGAGTCGCTTGC |
| mouse Nrf2-R | GCCAAACTTGCTCCATGTCC |
| mouse Fth1-F | ACAAAGATCGGGACTGCTAATGA |
| mouse Fth1-R | GAAACATCATCTCGGTCAAA |
| mouse Slc7a11-F | TGGCGGTGACCTTCTCTGA |
| mouse Slc7a11-R | ACAAAGATCGGGACTGCTAATGA |
| mouse Gclc-F | AACACAGACCCAACCCAGAG |
| mouse Gclc-R | CCGCATCTTCTGGAAATGTT |
| mouse Gclm-F | GCCACCAGATTTGACTGCCTTT |
| mouse Gclm-R | CAGGGATGCTTTCTTGAAGAGCTT |
| mouse Gpx4-F | GCCAAACTTGCTCCATGTCC |
| mouse Gpx4-R | GCCAAACTTGCTCCATGTCC |
| mouse Gapdh-F | GGCAAATTCAACGGCACA |
| mouse Gapdh-R | GTTAGTGGGGTCTCGCTCCTG |
